# Precision wings treating skeletal class II in growing patients: a systematic review and meta-analysis

**DOI:** 10.1186/s40510-025-00564-4

**Published:** 2025-05-26

**Authors:** Paulo Mecenas, Renata Travassos da Rosa Moreira Bastos, Nathalia Carolina Fernandes Fagundes, David Normando

**Affiliations:** 1 Federal University of Pará (UFPA), Belém, Brazil; 2 Pará State University Center, Belém, Brazil; 3 University of Alberta, Edmonton, Canada

**Keywords:** Invisalign, Clear aligner appliances, Angle class II, Mandibular retrusion, Mandibular advancement

## Abstract

**Background:**

Skeletal Class II malocclusion, often associated with mandibular deficiency, is commonly treated with functional appliances. Precision Wings are a functional appliance that provides an alternative approach by combining mandibular advancement with dental alignment.

**Objective:**

This systematic review aimed to evaluate the effectiveness of Precision Wings in treating skeletal Class II malocclusion in growing patients.

**Eligibility criteria:**

Studies assessing the correction of skeletal Class II malocclusion in growing patients treated with Precision Wings were selected according to the PICOS strategy. The PRISMA guidelines were followed.

**Information sources:**

Unrestricted electronic searches were conducted across seven databases up to February 2025.

**Risk of bias and synthesis of results:**

The ROBINS-I tool was used to assess the risk of bias (RoB) in non-randomized studies. A random-effects meta-analysis was performed, and the certainty of the evidence was evaluated using the GRADE approach.

**Results:**

Seven studies were included, and data were extracted. Mean differences (MDs) with 95% confidence intervals (CIs) were calculated using random-effects meta-analysis. The findings suggest that Precision Wings may be effective in treating skeletal Class II malocclusion in growing patients through both dental and skeletal changes. Regarding skeletal effects, sagittal changes were limited to the mandible and were of small magnitude, with a reduction in ANB° (MD = -0.81; 95% CI: -1.04 to -0.58; *p* < 0.001) occurring exclusively due to an increase in SNB° (MD = 0.55; 95% CI: 0.11 to 0.98; *p* = 0.01), while no significant changes were observed in SNA° (MD = -0.02; 95% CI: -0.42 to 0.38; *p* = 0.91). The included studies did not report significant vertical effects. Meta-analyses comparing Precision Wings with other functional appliances were not feasible due to the small number of studies evaluating each comparison and the substantial clinical and methodological heterogeneity across the included studies.

**Conclusion:**

Although the available scientific evidence on this topic is limited, treatment with Precision Wings appears to offer minimal clinical improvement in mandibular growth for the correction of skeletal Class II malocclusion. To obtain more conclusive findings, future research should prioritize well-structured randomized clinical trials with standardized treatment protocols, extended follow-ups, and consistent cephalometric assessment methods.

**Supplementary Information:**

The online version contains supplementary material available at 10.1186/s40510-025-00564-4.

## Introduction

### Rationale

Class II malocclusion is one of the most common orthodontic issues, characterized by various dental alterations that may or may not be associated with a maxillomandibular discrepancy [1, 2]. Among children in the mixed dentition stage, the global prevalence of Class II malocclusion is estimated to be 23% [3]. In Caucasian and Brazilian children, prevalence rates reported in different studies are 26% and 38%, respectively, ranking second only to Class I in frequency [4, 5].

The skeletal discrepancy in Class II malocclusion is typically characterized by maxillary prognathism, mandibular retrognathism, or a combination of both [2]. The literature indicates that approximately 80% of cases are associated with mandibular deficiency [5, 6]. Consequently, in patients where mandibular retrusion is the primary component of skeletal Class II, orthodontists often employ growth modification techniques to stimulate mandibular development [7]. Many clinicians use functional orthopedic appliances, such as the Herbst and Twin Block (TB), in growing patients with mandibular retrusion. These appliances maintain the mandible in an advanced position, stimulating condylar growth and leading to overjet reduction and Class II correction [2, 8].

While these traditional functional appliances have demonstrated effectiveness, patient compliance remains a challenge due to their bulkiness, discomfort, and negative aesthetic impact. From an early age, children can distinguish different types of smiles and develop certain aesthetic perceptions [9]. Consequently, clear aligners have gained prominence in recent years among this population due to advancements in polymeric materials and computer technology, which enhance comfort, aesthetics, and patient adherence to treatment [10].

In this category of appliances, Invisalign^®^ Precision Wings (Align Technology, Inc., San Jose, California) was introduced as an alternative for treating skeletal Class II malocclusion due to mandibular retrusion in patients in the late mixed or early permanent dentition. This system incorporates buccal projections on the upper and lower aligners, which interlock to hold the mandible in a more advanced position, thus facilitating both skeletal and dental modifications. Its primary advantage over traditional functional appliances is the ability to simultaneously promote mandibular advancement and tooth movement, facilitating both dental and skeletal changes while potentially reducing total treatment time [11–13].

### Objectives

Mandibular advancement with Precision Wings is a relatively recent treatment approach, and its long-term outcomes remain unclear in the scientific literature. Therefore, this systematic review and meta-analysis aims to assess the efficacy of Invisalign with Precision Wings in correcting skeletal Class II malocclusion by investigating the skeletal and dental changes in growing patients and comparing these outcomes to those achieved with clinically established functional orthopedic appliances.

## Materials and methods

### Protocol and registration

This systematic review was registered in the PROSPERO database (https://www.crd.york.ac.uk/prospero/) under the registration number CRD42024584232. The PRISMA guidelines were followed (Appendix 1) [14].

### Eligibility criteria

The inclusion criteria for study selection were based on the PICOS framework: Population– Growing patients with skeletal Class II malocclusion; Intervention– Treatment with Invisalign Precision Wings; Comparator– Treatment with a mandibular advancement appliance or no treatment; Outcomes– The primary outcomes were skeletal and dental sagittal cephalometric changes promoted by the treatment. Secondary outcomes included changes in vertical and soft tissue cephalometric variables. Study Design– Controlled clinical trials and observational studies.

Exclusion criteria included patients with craniofacial syndromes or anomalies, such as cleft lip and/or palate, surgical patients, individuals with periodontal or systemic diseases, tooth loss, or those treated with skeletal anchorage. Additionally, reviews, case reports, case series, opinion articles, conference abstracts, and book chapters were deemed ineligible.

### Information sources

Searches were conducted in seven electronic databases: PubMed, Scopus, Web of Science, EMBASE, the Cochrane Library, ProQuest, and the Trip Medical Database for gray literature (Appendix 2). No restrictions were applied regarding coverage dates or language. Additional relevant citations were identified through manual searches of the reference lists of all included articles. Efforts were made to identify potentially relevant unpublished or ongoing trials by contacting experts in the field. The searches were conducted in June 2024 and updated in February 2025.

### Search strategy and study selection

Searches were performed using controlled vocabulary and free-text terms related to the use of Invisalign and mandibular advancement in Class II treatment, adapted to the syntax rules of each bibliographic database (Appendix 2). All relevant citations were exported and saved in a reference manager (Rayyan software, Qatar Computing Research Institute, Doha, Qatar). Duplicate records were removed during the merging of all search results.

Study selection was conducted independently by two reviewers (P.M. and R.T.R.M.B.) in two phases. First, titles and abstracts were screened. In the second phase, full texts of potentially relevant studies were assessed. Any discrepancies were resolved through discussion, and when necessary, a third examiner (N.C.F.F.) was consulted to reach a final decision. Eligibility criteria were applied in both phases. If essential data were missing or unclear, the corresponding author of the study was contacted via email weekly for up to four consecutive weeks.

### Data extraction

Data were collected from the selected articles independently and in duplicate by the same authors (P.M. and R.T.R.M.B.). Extraction was performed manually using an Excel spreadsheet (Microsoft Excel, Microsoft Corporation, Redmond, Washington). When necessary, retrieved information was cross-checked with a third reviewer (N.C.F.F.). Extracted data included study details (author, year of publication, country, and study design), patient characteristics (sample size, sex, mean age per group, treatment or follow-up duration), results (skeletal, dental, and soft tissue cephalometric variables, and outcome measurement methods), and conclusions.

### Assessment of risk of bias in individual studies

The ROBINS-I tool was used to assess the risk of bias (RoB) in non-randomized studies of intervention [15]. Seven domains were evaluated: bias due to confounding, bias due to selection of participants, bias in classification of interventions, bias due to deviations from intended interventions, bias due to missing data, bias in measurement of outcomes, bias in selection of the reported results. The criteria for evaluating each domain are described in Table 1. The following responses were accepted: yes, probably yes, probably no, no, and not informed. Each domain was classified as having a low, moderate, serious, or critical risk of bias. Quality assessment judgments were not used as a threshold for study inclusion but rather as a potential explanation for differences in results. The risk of bias assessment was conducted by two previously calibrated reviewers, with disagreements resolved by a third reviewer if necessary. The reviewers used the ROBVIS (Risk of Bias Visualisation) tool to graphically present the results of the risk of bias assessment.

**Table 1:** Criteria adopted for risk of bias assessment using the ROBINS-I tool

Domains	Criteria
Bias due to confounding	Studies must account for the initial magnitude of Class II, gender, and age or growth stage as confounding factors.
Bias in classification of interventions	The process of diagnosing patients and administering treatment with Precision Wings should be reported in detail. The sample size calculation must also be accurate.
Bias in selection of participants into the study	Eligibility criteria must be clearly defined, and diagnostic methods must be efficient.
Bias due to deviations from intended interventions	Studies were evaluated regarding the clarity and consistency in applying the intervention protocol. When relevant details were missing or differences between the intended and actual interventions were likely, the risk of bias was considered higher.
Bias due to missing data	Large losses to follow-up, incomplete data collection, or the exclusion of participants from the analysis must be avoided.
Bias arising from measurement of the outcome	The correction of Class II malocclusion must be assessed using valid methods, and statistical analyses must be performed accurately.
Bias in selection of reported results	Selective reporting of the results when the effects of all measurements were not fully reported
Overall risk of bias	If at least one domain was identified as having a high risk of bias, the overall risk was classified as high. If at least one domain was of some concern but no domains were at high risk, the overall risk was classified as moderate. If all domains were at low risk of bias, the overall risk was classified as low.

### Summary of measurements and synthesis of results

The variations between untreated controls and Precision Wings were analyzed using Cochrane RevMan (Online Version: 8.6.0, Cochrane, London, UK) when the included studies exhibited methodological, statistical, and clinical consistency in data and collection methods. The mean difference with a 95% confidence interval was calculated. If any required data for the meta-analysis were missing, the study authors were contacted via email to request the necessary information.

Statistical heterogeneity was assessed using the I² index. Interpretation thresholds for the I² statistic were based on the Cochrane Handbook for Systematic Reviews of Interventions (www.training.cochrane.org/handbook; accessed September 4, 2024): 0–40%: may not be significant; 30–60%: might indicate moderate heterogeneity; 50–90%: could signify substantial heterogeneity; 75–100%: indicates considerable heterogeneity. Random-effects models were applied using the DerSimonian-Laird method, as the included studies were not functionally equivalent and the aim was to generalize the results of the meta-analysis [16].

### Certainty of evidence

The overall certainty of the evidence was evaluated using the Grading of Recommendations Assessment, Development, and Evaluation (GRADE) Pro software (https://www.gradepro.org/*)* [17]. Each of the five domains assessed in the included studies—study design, risk of bias, inconsistency, indirectness, and imprecision—was classified as having no serious, serious, or very serious concerns. However, three factors could increase the certainty of the evidence: the magnitude of the effect, the presence of a dose-response relationship, and the identification of potential confounders. The final classification was rated as high, moderate, low, or very low. A minimum difference of 1° in the angular variables was considered the threshold for potential clinical relevance, based on treatment effects reported in previous systematic reviews evaluating fixed and removable functional appliances in patients with Class II malocclusion.

## Results

### Study selection

**Fig. 1:**
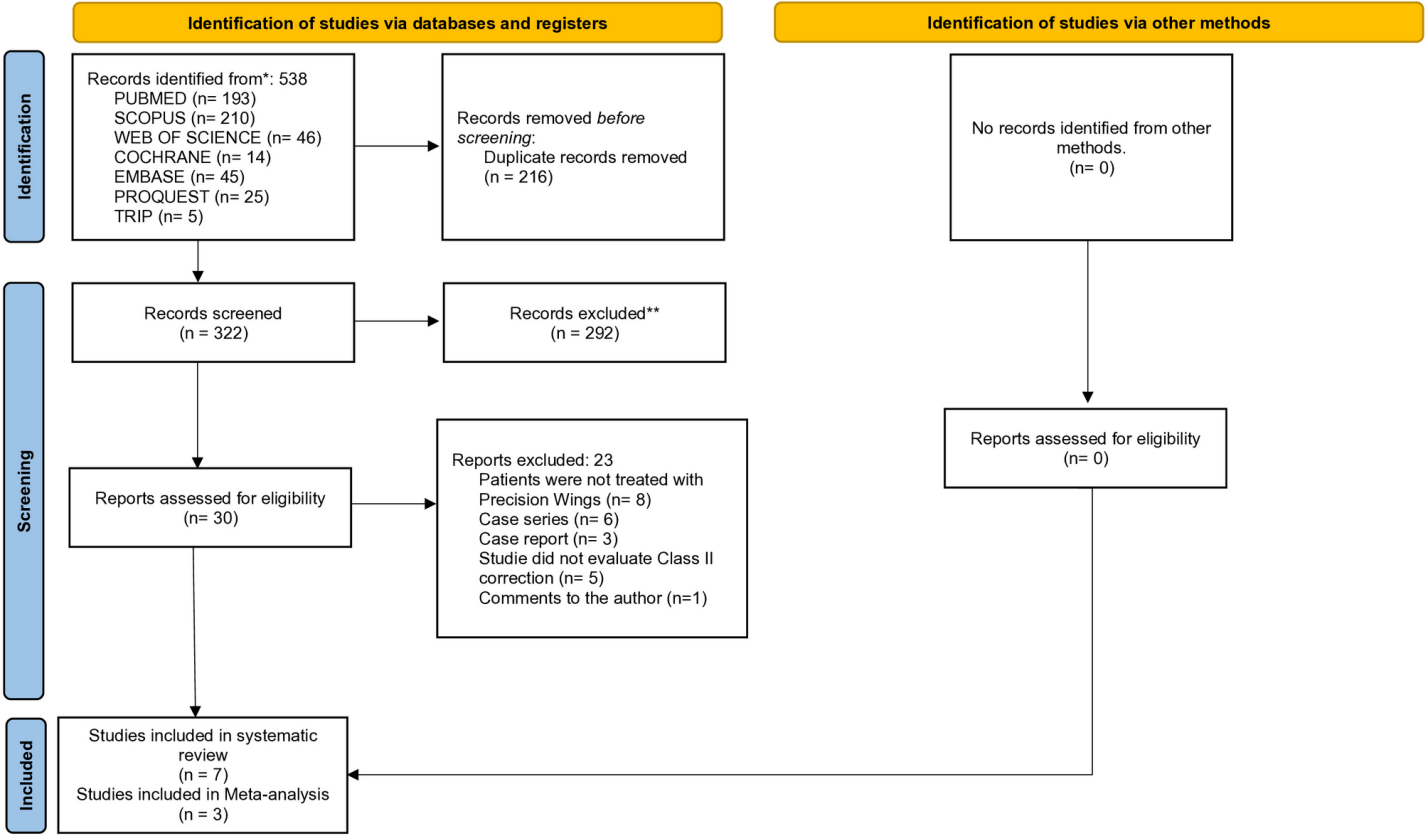
PRISMA flowchart of article retrieval

A total of 538 studies were retrieved from the electronic databases. After duplicate removal, the titles and abstracts of 322 articles were screened. Of these, 30 were reviewed in full (Appendix 3). Following this process, seven studies were included in the systematic review, with four selected for the meta-analysis (Fig. 1).

### Study characteristics

The characteristics of the seven included studies are summarized in Table 2. Among these manuscripts, six were classified as retrospective controlled clinical trials (CCTs) [12, 18–22], and one as a prospective CCT [23]. Participants were selected from private practices in three studies [12, 20, 23] and from university clinics in four [18, 19, 21, 22]. The sample size of the Precision Wings group ranged from 10 [19] to 50 [18], and participants’ ages varied from 9.7 ± 0.10 years [23] to 13.1 ± 1.5 years [20]. The Cervical Vertebral Maturation (CVM) method was not used to identify the growth stage by only one manuscript [18]. Individuals in stage CS2 were recruited in three studies [12, 20, 23], those in stage CS3 were included in five studies [19–23], and one study [20] included patients in stage CS4. An untreated control group was assessed in four studies [12, 18, 21, 23]. Treated control groups were also used: a TB group was evaluated in four manuscripts [12, 19, 21, 22], a Herbst group in two [12, 20], and a Vanbeek group in one [12].

**Table 2:** Characteristics of the included studies

1. Authorship	2. Material and methods			3. Results	4. Conclusion
Author, year, country and study design	Group, sample size (female/male), and mean age per group/ CVM stage	Follow-up duration/months (sample losses)	Aligners treatment protocol (mean number of aligners/aligners exchange period)	Outcomes measurement methods and evaluated variables	Main results
Al Subaie et al. 2024, Saudi Arabia, retrospective/ CCT [18]	UC: 20 (7/13), 11.75 ± 1.59. PW: 50 (27/23), 11.98 ± 2.18. The CVM stage was not specified.	UC: 19.2 ± 9.85. PW: 18.38 ± 5.05.	NI.	Lateral cephalograms were used to evaluate: *Skeletal variables*: - SNA, SNB, ANB, Wits, maxillary and mandibular base positions, A point N perpendicular to FH, Pog-N perpendicular distance, condylion to point A, condylion to gnathion, Go-Gn, Co-Go, mandibular plane angle, palatal plane. *Dental variables*: - Overjet, overbite, U1-PP, maxillary and mandibular incisors and molar position, IMPA. *Soft tissue variables*: - Cm-Sn-Ls1, G’-Sn-Pog’1, Sn-Gn’/c-Gn’ 1, Ls to E-plane-Pog’1, Li to E-plane-Pog1, Si to Li-Pog’1.	Treatment with PW led to an increase in SNB, mandibular base position, and Pog-N perpendicular distance compared to the UC group. Significant mesialization of mandibular molars and protrusion of lower incisors were observed, along with a marked reduction in overjet. Both groups showed upper lip retrusion, more pronounced in the PW group.lePara>
Caruso 2021, Italy, retrospective/ CCT [19]	TB: 10 (5/5), 10 ± 1.05. PW: 10 (5/5), 10 ± 1.05. CVM stage: CS3.	NI.	NI.	Lateral cephalograms were used to evaluate: *Skeletal cephalometric variables*: - SNA, SNB, ANB, Go-Me^ANSPNS, Ar-Go^Go-Me, FMA. *Dental cephalometric variables*: - U1^ANSPNS, L1^Go-Me.	Although both groups showed an increase in SNB, a greater reduction in ANB and overjet was observed in the TB group. Additionally, only the TB group showed a reduction in SNA and upper incisor retroinclination.
Elfouly et al. 2024, Egypt, retrospective CCT [22]	MB: 20 (9/11), 10.36 ± 1.04. TB: 20 (10/10), 10.56 ± 1.15. PW: 20 (11/9), 10.31 ± 1.16. CVM stage: CS3.	PW, MB and TB: monthly follow-ups until a Class I molar relationship was achieved.	Patients were required to wear their aligners for at least 22 h/day, which can be removed while eating. They were also notified to replace the aligners on a weekly basis according to their planned set of aligners.	Lateral cephalograms were used to evaluate: *Skeletal variables*: SNA, SNB, ANB, Wits, FMA.	No significant changes were found in SNA across groups. All appliances produced a significant increase in SNB and a reduction in ANB, with no intergroup differences. Wits appraisal also decreased significantly in all groups, but with no statistical difference among them. No significant vertical changes (FMA) were observed.
Hosseini et al. 2024, Canada, retrospective CCT [20]	Herbst: 20 (11/9), 12.7 ± 1.8. PW: 20 (11/9), 13.1 ± 1.5. CVM stage: CS2, CS3, and CS4.	Herbst: 18.5. PW: 14.4.	Patients in the Precision Wings group were treated until the molar was corrected to a Class I relationship on at least 1 buccal segment, or the overjet was edge-to-edge in centric relation position. During the advancement phase, the mandible was advanced sequentially 2 mm every 8 weeks until the incisors were edge-to-edge.	Lateral cephalograms were used to evaluate: *Skeletal cephalometric variables*: - SNA, SNB, ANB, Wits, SNL-NL, SNL-ML, SNL-OLs, OLp-Co, OLp-A-pt, OLp-B-pt, OLp-Pg, OLp-Gn, OLs-A-pt, ANS-Me, Msc-NL, Mic-ML. *Dental cephalometric variables*: - Overbite, OLp-Ms, OLp-Mi, OLp-ls, OLp-Ii, Is-NL, li-ML, ls/NL, li/ML.	Both groups showed similar changes in SNB, SNA, and ANB. However, the Herbst group showed greater reduction in overjet, molar relationship, Wits appraisal, and overbite. In contrast, the PW demonstrated superior control of mandibular incisor inclination.
Lombardo et al. 2023, Italy, retrospective CCT [21]	UC: 15 (11/4), 10.9 ± 1.1. TB: 35 (17/18), 12.0 ± 1.3. PW: 21 (12/9), 11.2 ± 1.1. CVM stage: CS3.	UC: 13.2 TB: 21.6. PW: 30.	Aligners were changed weekly. Patients were instructed to wear them a minimum of 22 h a day. Mean number of aligners not reported.	Lateral cephalograms were used to evaluate: *Skeletal variables*: - SNA, SNB, ANB, Wits, Co-Gn, SN-Pal.Pl., SN-GoGn (SN-Mand.Pl.), SpP-GoGn (Pal. Pl.-Mand. Pl.), CoGoMe, *Dental variables*: - Overjet, overbite, Upper Inc.-Pal.Pl., Lower Inc.-Mand. Pl. *Soft tissue variables*: - TVL-Pg’.	Both PW and TB showed similar reductions in ANB and increases in mandibular length. Overjet and overbite were reduced in both treated groups compared to UC. The TB group demonstrated greater advancement of the soft tissue pogonion.
Ravera et al. 2021, Italy, prospective CCT [23]	UC: CVM2–15, 9.2 ± 1.4; CVM3–17, 12.10 ± 1.4. PW: CVM2–20, 9.7 ± 0.10; CVM3–20, 11.8 ± 0.11. CVM stage: CS2 and CS3.	UC: (4 losses) CVM2–18. CVM3–17. PW: (3 losses) CVM2–18. CVM3–18.	NI	Lateral cephalograms were used to evaluate: *Skeletal cephalometric variables*: - SNA, SNB, ANB, Wits, SN-GoGn, SpP-GoGn, A-Downs, Pog, B-Downs, A-Pog, Co-Go, Co-Gn, Gn-Go. *Dental cephalometric variables*: - X11-SpP, X41-GoGn, X11-X41.	In the CS2 group, reductions were observed in ANB, A:Po, Wits index, and upper incisor inclination relative to the bispinal plane, along with advancement of the B Downs point and increased interincisal angle. In the CS3 group, improvements included Wits index reduction, increased SpP^GoGn angle, and greater mandibular length.
Wu et al. 2023, China, retrospective CCT [12]	UC: 12 (5/7), 10.41 ± 0.90. TB: 12 (5/7), 11.00 ± 1.04. Herbst: 11 (7/4), 11.55 ± 0.69. Vanbeek: 14 (7/7), 10.71 ± 1.44. PW: 14 (2/12), 12.11 ± 1.16. CVM stage: CS2.	UC: 10.25 ± 3.74. TB: 10.16 ± 5.46. Herbst: 10.18 ± 3.06. Vanbeek: 7.28 ± 2.30. PW: 22.84 ± 8.98.	NI	Lateral cephalograms were used to evaluate: *Skeletal cephalometric variables*: - SNA, SNB, ANB, mandibular plane angle (MP-FH), facial angle (FH-NP), angle of convexity (NA-PA), Co-Go, Go-Pog, Y-Axis angle, lower facial height ratio, vertical ratio (ALFH/PLFH), P-A face height (S-Go/N-Me). *Dental cephalometric variables*: - Maxillary incisor angle (U1-SN), mandibular incisor angle (L1-MP), occlusal plane angle (OP-FH), inter incisal angle (U1-L1), maxillary incisor to palatal plane (U1-PP), maxillary molar to palatal plane (U6-PP).	All treated groups showed an increase in SNB, resulting in a reduction in ANB compared to the UC. Co-Go values were greater in the TB and PW groups than in the Vanbeek and UC groups. Similarly, Co-Pog values were significantly higher in the Herbst, TB, and PW groups compared to UC, with TB and PW also showing significantly greater values than Vanbeek. The PW and TB groups demonstrated higher U1-PP values than the other groups. Regarding lower incisor inclination, the L1-MP measurement increased significantly in the Herbst group, exceeding the changes observed in PW, Vanbeek, and UC.

CCT: Controlled Clinical Trial; UCT: Uncontrolled Clinical Trial; Precision Wings: Invisalign clear aligners with mandibular advancer wings; UC: untreated control; TB: Twin Block; PW: Precision Wings; NI: not informed; CVM2: Cervical stage 2 of the Cervical Vertebra Maturation Degree; CVM3: Cervical stage 3 of the Cervical Vertebra Maturation Degree; MB: Myobrace.; UC: untreated control

The minimum follow-up period was 14.4 months [20], while the maximum was 30 months [21]. The treatment protocol for Precision Wings was well-documented in two studies [20, 21], partially described in one [22], and not mentioned in four [12, 18, 19, 23]. None of the manuscripts reported the mean number of aligners used. The aligner exchange period was described in three studies [20–22] and was seven days for all. Lateral cephalograms [12, 18–21, 23] were used in the included studies to assess the cephalometric variables.

### Risk of bias

**Fig. 2:**
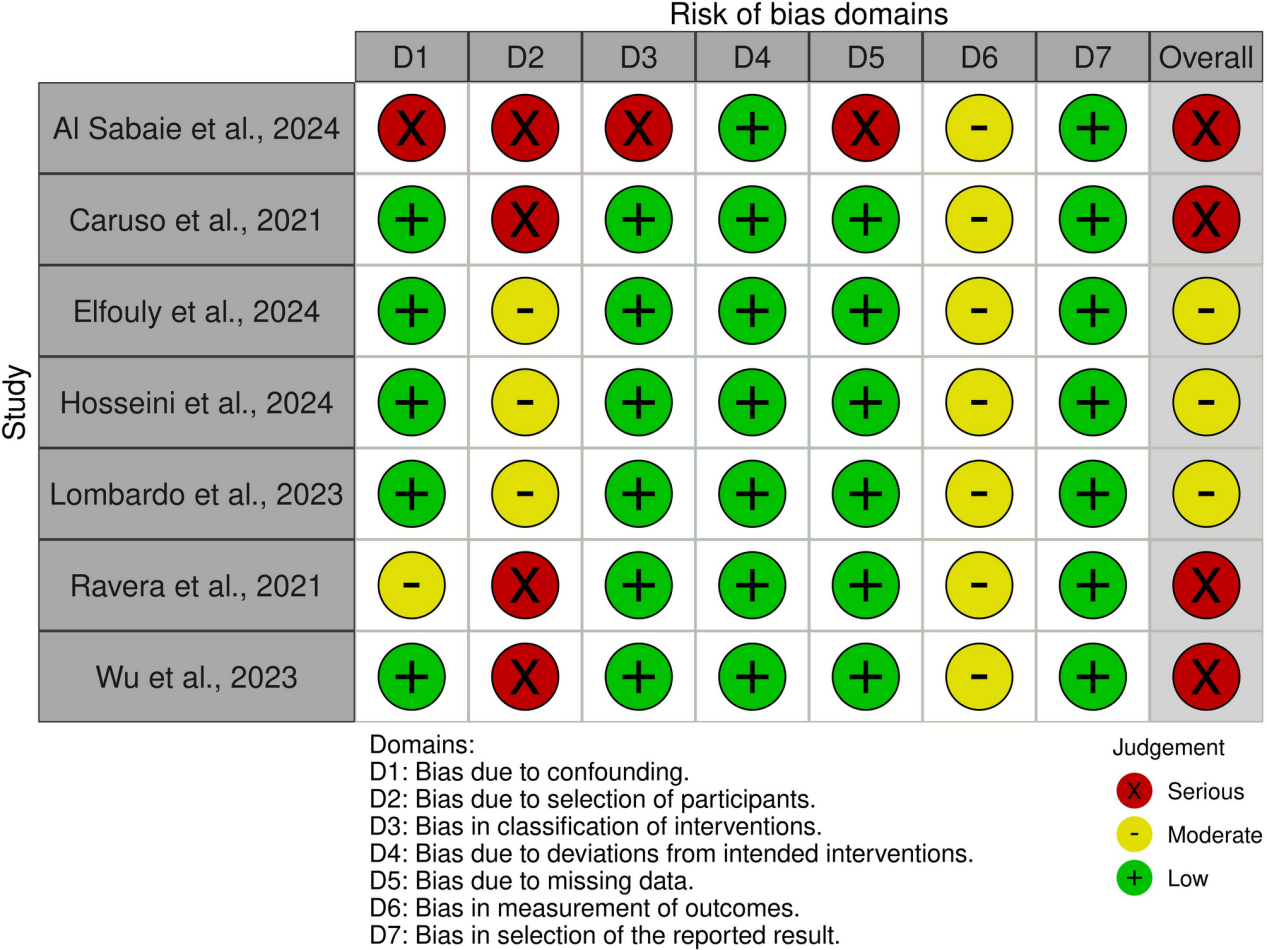
Risk of bias assessment using ROBINS-I tool

The ROBINS-I tool [15] was used to evaluate the risk of bias (RoB). The main sources of bias identified in the included studies were the lack of control for confounding factors, such as unclear criteria regarding the initial severity of Class II malocclusion and growth stage [18], or the absence of reported patient sex [23]. Issues related to the classification of interventions were also noted, including insufficient reporting or lack of description of the Precision Wings treatment protocol [12, 18, 19, 21–23]. Bias in the selection of participants into the study was observed in one article that did not use any method to assess the growth stage of the sample [18]. Missing data, due to the exclusion of cases without complete documentation, was another concern [18]. Additional sources of bias included differences in follow-up periods between groups [12, 20, 21] or the absence of follow-up duration reporting [19, 22]. Moreover, concerns were raised regarding the choice of statistical tests [12, 18–23], as more robust multivariate analyses, such as MANOVA or Hotelling’s T², should have been used to reduce the risk of inflated false-positive results. Four controlled clinical trials were judged to have a high RoB [12, 18, 19, 23], while the remaining three [20–22] were rated as having some concerns. A detailed assessment of RoB is presented in Fig. 2.

### Results of individual studies

Given the different comparisons between groups, these analyses will be presented separately. The first section focuses on the comparison between patients treated with Precision Wings and untreated controls. Subsequently, separate comparisons are made between the Precision Wings group and those treated with Twin Block, Herbst, or Van Beek appliances. The results of the main measures compared between the groups are summarized in the Appendix 4.

### Comparison between precision wings and untreated control

Regarding the main skeletal sagittal variables, no effects on SNA were observed in any included study. Two studies [21, 23] indicated no changes in SNB, whereas two others [12, 18] reported an increase in the Precision Wings group. All manuscripts [12, 18, 21, 23] documented a decrease in ANB among treated patients. A reduction in the Wits appraisal was observed in two studies [18, 23], while one manuscript [21] found no difference. The Co-Gn measurement increased in two papers [21, 23] and showed no difference in one [18]. Similarly, Go-Gn exhibited no significant changes in two studies [18, 23].

Concerning dental sagittal measures, no manuscript reported alterations in the mean IMPA [12, 18, 21, 23]. Two papers [18, 21] found no effects on U1-PP, while one study [23] observed lingual inclination, and another [12] identified buccal inclination of the upper incisors. Overjet reduction was noted in three studies [12, 18, 21]. Additionally, lower incisor protrusion and mesial movement of the lower molars were recorded in one paper [18].

Among the primary vertical cephalometric variables, no significant differences were identified between groups for SNGoGn [12, 18, 21, 23], Palatal Plane [18, 19, 21], SN-Pal.Pl [21], or CoGoMe [21]. Improvement in Co-Go was noted in one manuscript [12] within the Precision Wings group, although two other studies [18, 23] found no significant changes. Overbite remained unchanged in one study [18], whereas another [21] documented a reduction. Molar extrusion (U6-PP) was evaluated in a single manuscript [12], which identified this outcome in the Precision Wings group.

In terms of soft tissue assessment, no differences were observed in the nasolabial angle. However, a reduction in upper lip protrusion and an increase in lower lip protrusion were reported in the treated group [18]. The evaluation of TVL-Pg’ in one paper [21] indicated improvement in the Precision Wings group.

### Comparison between precision wings group and TB group

Four studies [12, 19, 21, 22] compared individuals treated with Precision Wings to those treated with the TB appliance. One study [12] did not report the standard deviation of the difference between pre- and post-treatment values when comparing the same group at the beginning and after treatment. Only the mean and p-value were described. Attempts were made to contact the corresponding author via email. After three attempts, the author responded and promised to send the missing data; however, it was never provided, and no further responses were received. Regarding sagittal measures, three manuscripts [12, 21, 22] reported no differences between the groups in SNA, while one study [19] found a reduction of this variable in the TB group. No differences were observed between the groups for SNB, which increased in all studies. Three papers [12, 21, 22] found no difference in ANB, whereas another manuscript [19] noted that the TB group showed a larger reduction in ANB compared to the Precision Wings group. Similarly, no differences were observed for Wits appraisal [21, 22], U1-PP [12, 21], IMPA [12, 19, 21], or Co-Go [12]. However, a greater overjet reduction was identified in the TB group in two studies [12, 19]. One paper [12] reported more pronounced skeletal effects than dental effects contributing to overjet correction in the TB group, while the Precision Wings group exhibited more dental effects than skeletal effects.

For vertical measures, no significant differences were reported for SNGoGn [12, 21, 23], FMA [19, 22], Co-Gn [21], or overbite [19, 21]. Regarding soft tissue variables, the only measure assessed was TVL-Pg’, which showed a greater increase in the TB group [21].

### Comparison between precision wings group and herbst group

Two manuscripts have compared the Precision Wings and Herbst groups [12, 20]. No significant effects on SNA were found, a similar increase in SNB, and a reduction in ANB in both groups, with no statistical differences between them. One study [20] reported a greater reduction in Wits appraisal in the Herbst group. For U1-PP, one manuscript observed minimal buccal inclination of upper incisors in the Precision Wings group [12], while another paper found no difference between the groups [20]. Both studies [12, 20] found an increase in the IMPA angle and a greater reduction in overjet in the Herbst group. One paper [12] reported that skeletal effects contributed more than dental effects to overjet reduction in the Herbst group, whereas the Precision Wings group showed a greater contribution from dental effects than skeletal effects.

Concerning the vertical effects, no statistical differences were reported in Co-Go [20], SNGoGn [12, 20], Is-NL [20], Msc-NL [20], and Mi-ML [20]. More overbite reduction in the Herbst group was found by one study [20], as well as intrusion of the lower incisors (Il-ML) [20] and maxillary molars [12]. Soft tissue effects of these therapies were not assessed.

### Comparison between precision wings group and Vanbeek group

Only one study [12] has compared individuals treated with Precision Wings to those treated with the Vanbeek appliance. In both groups, no treatment effect was observed on SNA, while a slight increase in SNB and a reduction in ANB were reported. The upper incisors exhibited buccal inclination in the Precision Wings group; in contrast, no changes were found in the Vanbeek group. The lower incisors showed no alterations in inclination in either group. A greater increase in Co-Go and Co-Pog was observed in the Precision Wings group, while no difference was reported for Go-Pog. Lower Facial Height Ratio, Vertical Ratio, and P-A Face Height also showed no differences between the groups. Individuals treated with Precision Wings exhibited more molar extrusion, represented by a greater increase in U6-PP. On the other hand, the Vanbeek appliance produced a greater reduction in overjet, with more skeletal than dental effects compared to the Precision Wings appliance.

### Data synthesis

**Fig. 3:**
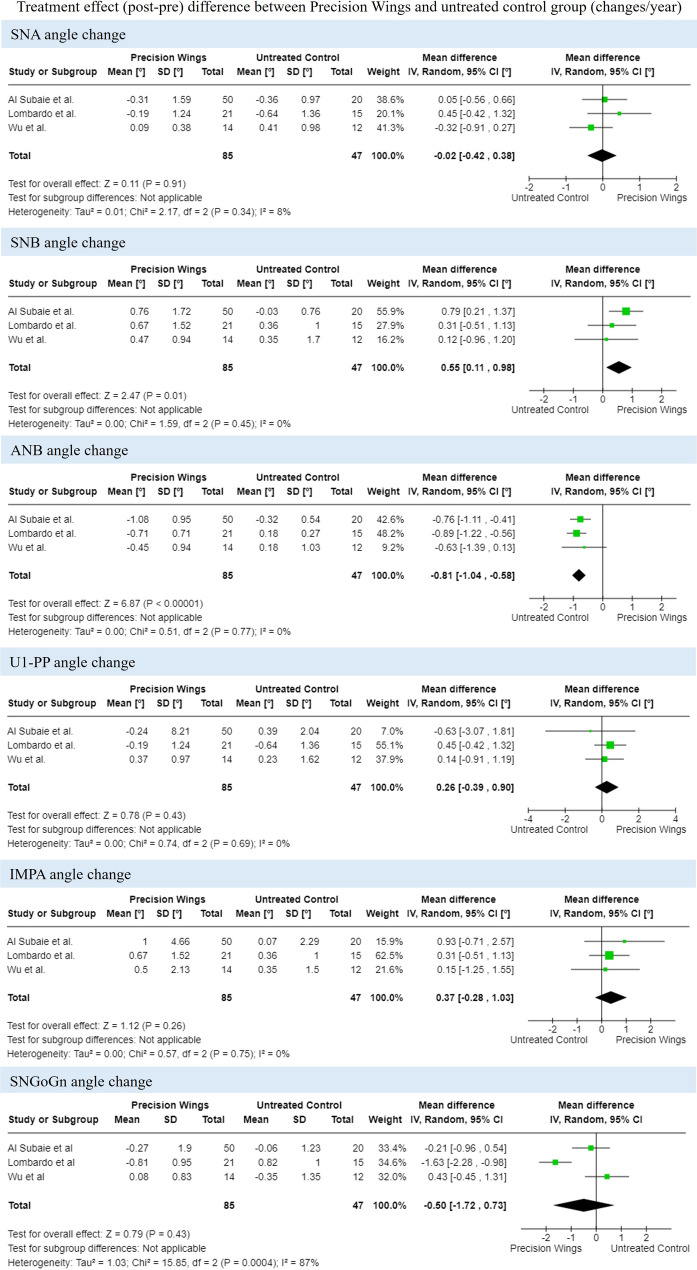
Random-effects meta-analysis of treatment effect differences between Precision Wings and untreated control group

Four papers compared the effects of Precision Wings treatment with those of an untreated control group; however, only three were included in the quantitative analysis [12, 18, 21]. One study [23] was excluded due to the absence of a description of the mean differences and standard deviations before and after treatment. Attempts were made to contact the corresponding authors of this manuscript via email. Emails were sent weekly for four consecutive weeks requesting the missing data. However, no response was received. Due to the limited number of included manuscripts, this meta-analysis can be characterized as an exploratory meta-analysis. A subgroup analysis based on CVM stage was not feasible, as it varied across the studies, and some of them included patients at multiple CVM stages within the same sample. The meta-analysis revealed no significant effects on SNA (three studies, MD = -0.02; 95% CI: -0.42 to 0.38; *p* = 0.91), but it showed a marginal increase in SNB (three studies, MD = 0.55; 95% CI: 0.11 to 0.98; *p* = 0.01), resulting in a minor decrease in ANB (three studies, MD = -0.81; 95% CI: -1.04 to -0.58; *p* < 0.001) in the Precision Wings group. U1-PPº (three studies, MD = 0.26; 95% CI: -0.39 to 0.90; *p* = 0.43), IMPAº (three studies, MD = 0.37; 95% CI: -0.28 to 1.03; *p* = 0.26), and SNGoGn (three studies, MD = -0.50; 95% CI: -1.72 to 0.73; *p* = 0.43) showed no significant difference (Fig. 3).

### Certainty of evidence

The certainty of evidence analyzed by GRADEpro was very low for all outcomes assessed. This was due to the nature of the studies evaluated, as all were non-randomized clinical trials, and their RoB. Sources of bias, such as the lack of definition of the initial Class II magnitude [18], the superficial description of the treatment protocol with Precision Wings [12, 18, 21], and inappropriate statistical analysis [12, 18, 21] impacted the certainty of evidence. Tables 3 and 4 illustrate the GRADEpro assessment.

**Table 3:** Summary of findings table according to the GRADE approach for precision wings versus untreated control

Outcomes	Anticipated absolute effects^*^ (95% CI)lePara>		No of participants (studies)	Certainty of the evidence (GRADE)	Comments
	Untreated Control	Difference in Precision Wings group			
SNA angle changes	The mean SNAº was − 0.19º	MD 0.02 º lower (0.42 lower to 0.38 higher)	132 (3 non-randomised studies)	⨁◯◯◯ Very low^a, b,c, d^	The treatment with Precision Wings has no effect on SNAº.
SNB angle changes	The mean SNBº was 0.22º	MD 0.55º higher (0.11 higher to 0.98 higher)	132 (3 non-randomised studies)	⨁◯◯◯ Very low^a, b,c, d^	Precision Wings treatment may result in a minimal increase in SNB°, with a mean change of 0.55°.
ANB angle changes	The mean ANBº was 0.01º	MD 0.81º lower (1.04 lower to 0.58 lower)	132 (3 non-randomised studies)	⨁◯◯◯ Very low^a, b,c, d^	Treatment with Precision Wings may slightly reduce ANB°, with an average change of 0.81°.
U1-PP angle changes	The mean U1-PPº was − 0.01º	MD 0.26º higher (0.39 lower to 0.90 higher)	132 (3 non-randomised studies)	⨁◯◯◯ Very low^a, b,c, d^	The treatment with Precision Wings has no effect on U1-PPº.
IMPA angle changes	The mean IMPAº was 0.26º	MD 0.37º higher (0.28 lower to 1.03 higher)	132 (3 non-randomised studies)	⨁◯◯◯ Very low^a, b,c, d^	Treatment with Precision Wings does not affect IMPAº.
SNGoGnº	The mean SNGoGn was 0,13º.	MD 0.5 lower (1.72 lower to 0.73 higher)	132 (3 non-randomised studies)	⨁◯◯◯ Very low^a, b,c, d^	The treatment with Precision Wings has no effect on SNGoGn.

a The initial Class II magnitude was not clearly defined (Al Subaie et al., 2024). b The treatment protocol with Precision Wings was not thoroughly described in the studies (Al Subaie et al., 2024; Lombardo et al., 2023; Wu et al., 2023). c Differences in the follow−up periods between groups were noted (Lombardo et al., 2023; Wu et al., 2023). d The statistical analyses performed were not the most appropriate (Al Subaie et al., 2024; Lombardo et al., 2023; Wu et al., 2023)

**Table 4:** Narrative gradepro approach for skeletal effects of precision wings

Certainty assessment							Impact	Certainty	Importance
No of studies	Study design	Risk of bias	Inconsistency	Indirectness	Imprecision	Other considerations			
Sagittal effects									
7	Non-randomised studies	Very serious^a, b,c^	Not serious	Not serious	Not serious	None	The sagittal effects achieved with Precision Wings treatment appear to fall below the minimal clinically important difference.	⨁◯◯◯ Very low^a, b,c^	CRITICAL
Vertical effects									
7	Non-randomised studies	Very serious^a, b,c^	Not serious	Not serious	Not serious	None	No significant effects of Precision Wings treatment were observed on most variables assessing vertical changes, regardless of comparison with the untreated control group, the TB group, or the Herbst group.	⨁◯◯◯ Very low^a, b,c^	CRITICAL
Soft tissue effects									
2	non-randomised studies	Very serious^a, b,c^	Not serious	Not serious	Not serious	None	Only two studies evaluated soft tissue changes. Some improvements in upper and lower lip protrusion, as well as in TVL-Pg’, were reported in the group treated with Precision Wings.	⨁◯◯◯ Very low^a, b,c^	CRITICAL

^a^The study did not thoroughly describe the protocol of treatment with Precision Wings (Wu et al., 2023); ^b^Difference in the follow-up period between groups (Hosseini et al., 2024; Wu et al., 2023); ^c^Difference in the follow-up period between groups (Hosseini et al., 2024; Lombardo et al., 2023; Wu et al., 2023); ^d^The study did not perform the most appropriate statistical analysis (Hosseini et al., 2024; Wu et al., 2023)

## Discussion

Despite the limitations of the selected manuscripts, the findings of this systematic review suggest that Precision Wings may contribute to the correction of skeletal Class II malocclusion in growing patients, primarily through modest effects. Among the skeletal changes, sagittal effects were limited to the mandible, with a slight reduction in ANB° resulting exclusively from a minimal increase in SNB°, while no significant changes were observed in SNA°. The inclination of the incisors relative to their bony bases did not demonstrate any statistically significant changes. Additionally, overjet correction achieved with Precision Wings appeared less effective than that reported for TB and Herbst appliances.

Functional appliances, such as the TB, Bionator, and Frankel appliance, have demonstrated some skeletal effects in correcting Class II malocclusion in growing patients [24]. Regarding sagittal effects, therapy with these appliances was associated with a minimal reduction in SNA (11 studies; MD = − 0.28 degrees/year, 95% CI: −0.44 to − 0.12 degrees/year), a slight increase in SNB° (11 studies; MD = 0.62 degrees/year, 95% CI: 0.36–0.88 degrees/year), and a small decrease in ANB° (10 studies; MD = − 1.14 degrees/year, 95% CI: −1.52 to − 0.77 degrees/year) compared to untreated patients. In contrast, fixed functional appliances [25] were found to induce a small reduction in SNA (9 studies; MD = − 0.83 degrees/year, 95% CI: −1.17 to − 0.48), a slight increase in SNB (9 studies; MD = 0.87 degrees/year, 95% CI: 0.30–1.43), and a moderate decrease in ANB (9 studies; MD = − 1.74 degrees/year, 95% CI: −2.50 to − 0.98) compared to untreated Class II patients. As a fixed appliance, the effects observed were greater than those found with removable appliances and those reported in this present systematic review. Our meta-analysis reveals that Precision Wings treatment had no impact on SNA (three studies; MD = − 0.02; 95% CI: −0.42 to 0.38; *p* = 0.91), a slight improvement in SNB (three studies; MD = 0.55; 95% CI: 0.11 to 0.98; *p* = 0.01), and a small reduction in ANB (three studies; MD = − 0.81; 95% CI: −1.04 to − 0.58; *p* < 0.001). Therefore, in general, the observed changes have limited clinical significance. Despite reaching statistical significance, the Precision Wings group showed a change of less than 1° in SNB° and ANB° compared to the untreated control, which is unlikely to result in a clinically meaningful skeletal improvement.

Twin Block and Precision Wings are appliances that work by using inclined planes to advance the mandible, leading to neuromuscular adaptation and a new spatial arrangement. This forward positioning of the mandible triggers a series of changes that help correct mandibular retrusion and alter the cranio-cervical posture associated with Class II malocclusion [26]. In contrast, the Herbst appliance rigidly connects the first maxillary molar to the lower first bicuspid on both sides through a telescopic rod-and-tube mechanism, permanently pushing the mandible into a protruded position [27]. As a result, effects in the maxilla after treatment with Herbst, such as molar distalization and a reduction in SNA, are understandable and referred to as the “Headgear effect of the Herbst appliance” by Pancherz in 1993 [28]. The reduction in SNA° observed in the maxilla after treatment with removable [24] and fixed appliances [25] was not noted in the group treated with Precision Wings in the present systematic review. Instead, an increase in SNB and a subsequent reduction in ANB were observed in this group. That said, it is important to highlight that the skeletal changes promoted by Precision Wings were of smaller magnitude than those typically reported with both removable and fixed functional appliances. These findings suggest that, although Precision Wings may contribute to skeletal improvement, their impact appears to be more limited when compared to traditional functional devices [24, 25].

Among the dental effects, Precision Wings appears to provide better control of incisor inclination during treatment compared to TB and Herbst. In turn, one study reported fewer dental effects in patients treated with the Vanbeek appliance than in those treated with Precision Wings [12]. IMPA showed no significant changes in individuals treated with Precision Wings [12, 18, 21, 23]. Regarding U1-PP, two included studies [18, 21] reported no changes in this group, while one found lingual inclination [23], and another observed buccal inclination [12] of the upper incisors. The meta-analysis revealed that, compared to the untreated control group, Precision Wings treatment resulted in no significant changes in U1-PP° (three studies; MD = 0.33; 95% CI: -0.56 to 1.22; *p* = 0.47) or IMPA (three studies; MD = 0.99; 95% CI: -0.12 to 2.10; *p* = 0.08). This control of incisor inclination may be attributed to the structure of the aligner, which allows complete coverage of the dental crown.

Conversely, patients treated with TB, Herbst, or Vanbeek exhibited a greater reduction in overjet, likely due to a combination of more pronounced skeletal effects and increased incisor inclination observed with these appliances. One included study [12] reported that skeletal effects contributed more prominently to overjet correction in the TB, Herbst, and Vanbeek groups, whereas in the Precision Wings group, dental effects accounted for a higher proportion (51.09%) compared to skeletal effects (48.91%).

It is also relevant to highlight that the meta-analyses in this review included only three studies in each comparison, which may impact the power of the analysis. Additionally, all manuscripts were non-randomized and presented several issues in the RoB analysis. As a result, any conclusions drawn must be approached with caution. This is further supported by the GRADE assessment, which indicated low certainty of evidence for all outcomes assessed.

Only two studies have evaluated the effects of Precision Wings therapy on soft tissue. One of these [18] reported no difference between the group treated with Precision Wings and the untreated control group in terms of facial convexity angle°, nasolabial angle°, and mentolabial sulcus depth. However, reductions in upper and lower lip protrusion were observed. Another study [21] reported an improvement in TVL-Pg’ in individuals treated with Precision Wings compared to the untreated control group, although the effect was smaller than that observed in the TB group.

### Limitations and strengths

One limitation of this systematic review is the low quality of the included studies. All selected manuscripts were non-randomized clinical trials, a study design that complicates the control of potential confounding factors. Reliable randomization ensures that the groups being studied have similar prognoses at the start of the study. In non-randomized studies, even if the groups do not show differences in their initial characteristics, there is no guarantee that individuals will exhibit similar growth patterns. Since this review involves growing patients, their facial growth may have directly influenced the study results.

The included manuscripts also failed to report specific usage protocols for Precision Wings. Many studies did not clearly describe the treatment protocol for this device, which likely contributed to both methodological and clinical heterogeneity. One aspect of clinical heterogeneity was the variation in the growth stages of participants, ranging from CS1 to CS4. Additionally, differences in treatment or observation durations across studies may have influenced the outcomes, increasing the risk of bias and ultimately reducing the certainty of the evidence. Furthermore, the use of different cephalometric analyses to measure similar parameters added to the methodological variability.

Another methodological limitation is the magnification bias inherent to cephalometric analyses, particularly for linear measurements. The use of different radiographic equipment in the included studies may have introduced variations in magnification, potentially affecting the comparability of linear cephalometric variables across studies. However, angular measurements are generally unaffected by this bias.

To minimize the impact of these sources of heterogeneity on the meta-analyses, studies were selected in which patients had similar growth stages and common cephalometric variables. Additionally, a random-effects meta-analysis was performed to account for methodological and clinical variability across the included studies, providing a more generalized estimation of treatment effects.

Despite these limitations, the findings of this review are highly relevant, as they summarize the current literature on this topic and highlight the need for well-designed prospective studies in this area. The clinical application of Precision Wings has advanced faster than its comprehensive scientific validation. In this context, our systematic review provides valuable evidence by consolidating available data and critically assessing the effectiveness of this treatment approach. Additionally, despite the methodological constraints identified, we implemented measures to mitigate their impact on the results, such as selecting studies with similar growth stages and common cephalometric variables and performing a random-effects meta-analysis to account for clinical and methodological variability. By addressing these challenges, this study offers a structured and evidence-based perspective on the current state of knowledge, contributing to informed clinical decision-making and future research directions.

## Conclusion

Although the available scientific evidence on this topic is limited, treatment with Precision Wings appears to promote minimal clinical improvement in mandibular growth for the correction of skeletal Class II malocclusion. To provide more definitive conclusions, future research should focus on well-designed randomized clinical trials with standardized treatment protocols, long-term follow-ups, and consistent cephalometric evaluation methods.

## Electronic supplementary material

Below is the link to the electronic supplementary material.

Supplementary Material 1.

Supplementary Material 2.

Supplementary Material 3.

Supplementary Material 4.

## Data Availability

No datasets were generated or analysed during the current study.
