# Supplementary material for: Precision wings treating skeletal class II in growing patients: a systematic review and meta-analysis
Source: Prog Orthod. 2025 May 26;26:16. doi: 10.1186/s40510-025-00564-4 (PMC12104119; doi:10.1186/s40510-025-00564-4)
Supplement: Supplementary file 2 — Supplementary Material 2. [file 40510_2025_564_MOESM2_ESM.docx]

| **Appendix S2.** Search strategies runned out in each electronic database. | | |
| --- | --- | --- |
| **Database** | **Search Strategy** | **N** |
| **PUBMED** | ((((((((((((((((((((((((((((Mandibular Advancement[MeSH Terms]) OR (Mandibular Advancement[Title/Abstract])) OR (Advancement, Mandibular[Title/Abstract])) OR (Mandibular Retrusion[Title/Abstract])) OR (Mandibular Retrusions[Title/Abstract])) OR (Retrusion, Mandibular[Title/Abstract])) OR (Retrusions, Mandibular[Title/Abstract])) OR (Mandibular Retroposition[Title/Abstract])) OR (Mandibular Retropositions[Title/Abstract])) OR (Retroposition, Mandibular[Title/Abstract])) OR (Retropositions, Mandibular[Title/Abstract])) OR (Mandibular retrognathism[Title/Abstract])) OR (Mandibular retraction[Title/Abstract])) OR (Mandibular retrognathia[Title/Abstract])) OR (Malocclusion, Angle Class II[MeSH Terms])) OR (Malocclusion, Angle Class II[Title/Abstract])) OR (Angle Class II[Title/Abstract])) OR (Class II, Angle[Title/Abstract])) OR (Malocclusion, Angle Class II, Division 2[Title/Abstract])) OR (Angle Class II, Division 2[Title/Abstract])) OR (Class II Malocclusion, Division 2[Title/Abstract])) OR (Malocclusion, Angle Class II, Division 1[Title/Abstract])) OR (Angle Class II, Division 1[Title/Abstract])) OR (Class II Malocclusion, Division 1[Title/Abstract])) OR (Skeletal class II[Title/Abstract])) OR (Retrognathia[MeSH Terms])) OR (Retrognathia[Title/Abstract])) OR (Retrognathias[Title/Abstract])) AND (((((((((((((((((((((((((Clear Aligner Appliances[Title/Abstract]) OR (Aligner Appliance, Clear[Title/Abstract])) OR (Aligner Appliances, Clear[Title/Abstract])) OR (Appliance, Clear Aligner[Title/Abstract])) OR (Appliances, Clear Aligner[Title/Abstract])) OR (Clear Aligner Appliance[Title/Abstract])) OR (Clear Dental Braces[Title/Abstract])) OR (Brace, Clear Dental[Title/Abstract])) OR (Braces, Clear Dental[Title/Abstract])) OR (Clear Dental Brace[Title/Abstract])) OR (Invisalign[Title/Abstract])) OR (Precision wings[Title/Abstract])) OR (MAA aligners[Title/Abstract])) OR (MA aligners[Title/Abstract])) OR (Mandibular wings[Title/Abstract])) OR (IAMW[Title/Abstract])) OR (Clear aligner therapy[Title/Abstract])) OR (CAT[Title/Abstract])) OR (Clear aligner[Title/Abstract])) OR (Mandibular leading appliance[Title/Abstract])) OR (Invisible aligner[Title/Abstract])) OR (Invisible braces[Title/Abstract])) OR (Invisalign MA[Title/Abstract])) OR (Invisible functional appliances[Title/Abstract])) OR (Invisible mandibular leading appliance[Title/Abstract])) | 193 |
| **SCOPUS** | TITLE-ABS-KEY ( ( "Mandibular Advancement" OR "Advancement, Mandibular" OR retrognathia OR retrognathias OR "Mandibular Retrusion" OR "Mandibular Retrusions" OR "Retrusion, Mandibular" OR "Retrusions, Mandibular" OR "Mandibular Retroposition" OR "Mandibular Retropositions" OR "Retroposition, Mandibular" OR "Retropositions, Mandibular" OR "Mandibular retrognathism" OR "Mandibular retraction" OR "Mandibular retrognathia" OR "Malocclusion, Angle Class II" OR "Angle Class II" OR "Class II, Angle" OR "Malocclusion, Angle Class II, Division 2" OR "Angle Class II, Division 2" OR "Class II Malocclusion, Division 2" OR "Malocclusion, Angle Class II, Division 1" OR "Angle Class II, Division 1" OR "Class II Malocclusion, Division 1" OR "Skeletal class II" ) AND ( "Clear Aligner Appliances" OR "Aligner Appliance, Clear" OR "Aligner Appliances, Clear" OR "Appliance, Clear Aligner" OR "Appliances, Clear Aligner" OR "Clear Aligner Appliance" OR "Clear Dental Braces" OR "Brace, Clear Dental" OR "Braces, Clear Dental" OR "Clear Dental Brace" OR invisalign OR "Precision wings" OR "MAA aligners" OR "MA aligners" OR "Mandibular wings" OR iamw OR "Clear aligner therapy" OR cat OR "Clear aligner" OR "Mandibular leading appliance" OR "Invisible aligner" OR "Invisible braces" OR "Invisalign MA" OR "Invisible functional appliances" OR "Invisible mandibular leading appliance" ) ) | 210 |
| **WEB OF SCIENCE** | TS=((“Mandibular Advancement” OR “Advancement, Mandibular” OR Retrognathia OR Retrognathias OR “Mandibular Retrusion” OR “Mandibular Retrusions” OR “Retrusion, Mandibular” OR “Retrusions, Mandibular” OR “Mandibular Retroposition” OR “Mandibular Retropositions” OR “Retroposition, Mandibular” OR “Retropositions, Mandibular” OR “Mandibular retrognathism” OR “Mandibular retraction” OR “Mandibular retrognathia” OR “Malocclusion, Angle Class II” OR “Angle Class II” OR “Class II, Angle” OR “Malocclusion, Angle Class II, Division 2” OR “Angle Class II, Division 2” OR “Class II Malocclusion, Division 2” OR “Malocclusion, Angle Class II, Division 1” OR “Angle Class II, Division 1” OR “Class II Malocclusion, Division 1” OR “Skeletal class II”) AND (“Clear Aligner Appliances” OR “Aligner Appliance, Clear” OR “Aligner Appliances, Clear” OR “Appliance, Clear Aligner” OR “Appliances, Clear Aligner” OR “Clear Aligner Appliance” OR “Clear Dental Braces” OR “Brace, Clear Dental” OR “Braces, Clear Dental” OR “Clear Dental Brace” OR Invisalign OR “Precision wings” OR “MAA aligners” OR “MA aligners” OR “Mandibular wings” OR IAMW OR “Clear aligner therapy” OR CAT OR “Clear aligner” OR “Mandibular leading appliance” OR “Invisible aligner” OR “Invisible braces” OR “Invisalign MA” OR “Invisible functional appliances” OR “Invisible mandibular leading appliance”)) | 46 |
| **COCHRANE** | (“Mandibular Advancement” OR “Advancement, Mandibular” OR Retrognathia OR Retrognathias OR “Mandibular Retrusion” OR “Mandibular Retrusions” OR “Retrusion, Mandibular” OR “Retrusions, Mandibular” OR “Mandibular Retroposition” OR “Mandibular Retropositions” OR “Retroposition, Mandibular” OR “Retropositions, Mandibular” OR “Mandibular retrognathism” OR “Mandibular retraction” OR “Mandibular retrognathia” OR “Malocclusion, Angle Class II” OR “Angle Class II” OR “Class II, Angle” OR “Malocclusion, Angle Class II, Division 2” OR “Angle Class II, Division 2” OR “Class II Malocclusion, Division 2” OR “Malocclusion, Angle Class II, Division 1” OR “Angle Class II, Division 1” OR “Class II Malocclusion, Division 1” OR “Skeletal class II”) AND (“Clear Aligner Appliances” OR “Aligner Appliance, Clear” OR “Aligner Appliances, Clear” OR “Appliance, Clear Aligner” OR “Appliances, Clear Aligner” OR “Clear Aligner Appliance” OR “Clear Dental Braces” OR “Brace, Clear Dental” OR “Braces, Clear Dental” OR “Clear Dental Brace” OR Invisalign OR “Precision wings” OR “MAA aligners” OR “MA aligners” OR “Mandibular wings” OR IAMW OR “Clear aligner therapy” OR CAT OR “Clear aligner” OR “Mandibular leading appliance” OR “Invisible aligner” OR “Invisible braces” OR “Invisalign MA” OR “Invisible functional appliances” OR “Invisible mandibular leading appliance”) in Title Abstract Keyword - (Word variations have been searched) | 14 |
| **EMBASE** | 'mandibular advancement':ti,ab,kw OR 'advancement, mandibular':ti,ab,kw OR retrognathia:ti,ab,kw OR retrognathias:ti,ab,kw OR 'mandibular retrusion':ti,ab,kw OR 'mandibular retrusions':ti,ab,kw OR 'retrusion, mandibular':ti,ab,kw OR 'retrusions, mandibular':ti,ab,kw OR 'mandibular retroposition':ti,ab,kw OR 'mandibular retropositions':ti,ab,kw OR 'retroposition, mandibular':ti,ab,kw OR 'retropositions, mandibular':ti,ab,kw OR 'mandibular retrognathism':ti,ab,kw OR 'mandibular retraction':ti,ab,kw OR 'mandibular retrognathia':ti,ab,kw OR 'malocclusion, angle class ii':ti,ab,kw OR 'angle class ii':ti,ab,kw OR 'class ii, angle':ti,ab,kw OR 'malocclusion, angle class ii, division 2':ti,ab,kw OR 'angle class ii, division 2':ti,ab,kw OR 'class ii malocclusion, division 2':ti,ab,kw OR 'malocclusion, angle class ii, division 1':ti,ab,kw OR 'angle class ii, division 1':ti,ab,kw OR 'class ii malocclusion, division 1':ti,ab,kw OR 'skeletal class ii':ti,ab,kw AND 'clear aligner appliances':ti,ab,kw OR 'aligner appliance, clear':ti,ab,kw OR 'aligner appliances, clear':ti,ab,kw OR 'appliance, clear aligner':ti,ab,kw OR 'appliances, clear aligner':ti,ab,kw OR 'clear aligner appliance':ti,ab,kw OR 'clear dental braces':ti,ab,kw OR 'brace, clear dental':ti,ab,kw OR 'braces, clear dental':ti,ab,kw OR 'clear dental brace':ti,ab,kw OR invisalign:ti,ab,kw OR 'precision wings':ti,ab,kw OR 'maa aligners':ti,ab,kw OR 'ma aligners':ti,ab,kw OR 'mandibular wings':ti,ab,kw OR iamw:ti,ab,kw OR 'clear aligner therapy':ti,ab,kw OR cat:ti,ab,kw OR 'clear aligner':ti,ab,kw OR 'mandibular leading appliance':ti,ab,kw OR 'invisible aligner':ti,ab,kw OR 'invisible braces':ti,ab,kw OR 'invisalign ma':ti,ab,kw OR 'invisible functional appliances':ti,ab,kw OR 'invisible mandibular leading appliance':ti,ab,kw | 45 |
| **PROQUEST** | "Precision wings" and "class II" | 25 |
| **TRIP** | “Mandibular advancement” and Invisalign | 5 |
