# Supplementary material for: Precision wings treating skeletal class II in growing patients: a systematic review and meta-analysis
Source: Prog Orthod. 2025 May 26;26:16. doi: 10.1186/s40510-025-00564-4 (PMC12104119; doi:10.1186/s40510-025-00564-4)
Supplement: Supplementary file 3 — Supplementary Material 3. [file 40510_2025_564_MOESM3_ESM.docx]

| **Appendix 3.** Studies identified from the literature search with their inclusion / exclusion status (with reasons). | | |
| --- | --- | --- |
| **Paper** | **inclusion/exclusion** | **by** |
| Pichler, E. and S. Scheibenreiter (1973). "[3 children with crying cat syndrome]." Wien Med Wochenschr 123(1): 19-21. | excluded/irrelevant | title |
| Schendel, S. A., et al. (2013). "3-dimensional facial simulation in orthognathic surgery: Is it accurate?" Journal of Oral and Maxillofacial Surgery 71(8): 1406-1414. | excluded/irrelevant | title |
| Fraccaro, M., et al. (1980). "The 11q-22q translocation - a european collaborative analysis of 43 cases." Human Genetics 56(1): 21-51. | excluded/irrelevant | title |
| Ojima, K., et al. (2014). "Accelerated extraction treatment with Invisalign." J Clin Orthod 48(8): 487-499. | excluded/irrelevant | title |
| Liebregts, J., et al. (2015). "Accuracy of three-dimensional soft tissue simulation in bimaxillary osteotomies." Journal of Cranio-Maxillofacial Surgery 43(3): 329-335. | excluded/irrelevant | title |
| McMorrow, S. M. and D. T. Millett (2017). "Adult orthodontics in the Republic of Ireland: specialist orthodontists’ opinions." Journal of Orthodontics 44(4): 277-286. | excluded/irrelevant | title |
| Staderini, E., et al. (2022). "Analysis of the Changes in Occlusal Plane Inclination in a Class II Deep Bite "Teen" Patient Treated with Clear Aligners: A Case Report." Int J Environ Res Public Health 19(2). | excluded/case report | title |
| Miyatani, N., et al. (2018). "Anesthetic Management of a Patient with 5p-Syndrome." Journal of Japanese Dental Society of Anesthesiology 46(1): 49-51. | excluded/irrelevant | title |
| Beri, A., et al. (2023). "Appliances Therapy in Obstructive Sleep Apnoea: A Systematic Review and Meta-Analysis." Cureus 15(11): e48280. | excluded/irrelevant | title |
| Al Subaie, H., et al. (2024). "Assessment of dental, skeletal, and soft tissue changes following mandibular advancement with Invisalign in skeletal Class II." Saudi Dent J 36(1): 66-71. | included |  |
| Rodríguez-Caballero, A., et al. (2012). "Assessment of orofacial characteristics and oral pathology associated with cri-du-chat syndrome." Oral Diseases 18(2): 191-197. | excluded/irrelevant | title |
| Ovard, S., et al. (2023). "Bias in the superimposition of lateral cephalograms in adult patients with anterior open bite." Am J Orthod Dentofacial Orthop 163(2): 222-232.e222. | excluded/irrelevant | title |
| Guo, R., et al. (2024). "Biomechanical analysis of miniscrew-assisted molar distalization with clear aligners: a three-dimensional finite element study." Eur J Orthod 46(1). | excluded/irrelevant | title |
| Wang, Y., et al. (2024). "Biomechanical effects of different mandibular movements and torque compensations during mandibular advancement with clear aligners: a finite element analysis." Front Bioeng Biotechnol 12: 1496517. | excluded/irrelevant | title |
| Zhang, M. X., et al. (2024). "Biomechanical effects of functional clear aligners on the stomatognathic system in teens with class II malocclusion: a new model through finite element analysis." BMC Oral Health 24(1). | excluded/irrelevant | title |
| Sander, F. G. and A. Weinreich (1991). "[The bite-jumping-appliance]." Dtsch Stomatol (1990) 41(6): 195-198. | excluded/irrelevant | title |
| Segnini, C., et al. (2025). "CAD-based functional therapy during aligner treatment – the “En-Nova”-protocol (technical report)." Seminars in Orthodontics 31(1): 110-114. | excluded/irrelevant | title |
| Yang, Y., et al. (2024). "Cantilever-aided bodily protraction of a mandibular molar with clear aligner: A finite element analysis." Int Orthod 22(4): 100924. | excluded/irrelevant | title |
| Dhanasekaran, M., et al. (2021). "Case report An esthetic approach to treat class II subdivision malocclusion using clear aligners." European Journal of Molecular and Clinical Medicine 8(3): 3126-3132. | excluded/case report | title |
| (2021). "A case report of an adolescent patient with skeletal mandibular retrognathia treated with invisible functional appliance." Chinese Journal of Orthodontics 28(4): 208-211. | excluded/case report | title |
| Kondo, E. and T. J. Aoba (1999). "Case report of malocclusion with abnormal head posture and TMJ symptoms." Am J Orthod Dentofacial Orthop 116(5): 481-493. | excluded/case report | title |
| Mascho, K., et al. (2024). "Case Report: An association of left ventricular outflow tract obstruction with 5p deletions." Frontiers in Genetics 15. | excluded/case report | title |
| Keller, K., et al. (2016). "CAT (Critically Appraised Topics) of the Month: Orthodontic Treatment of Class II Malocclusion During Early Childhood Does Not Produce Better Clinical Outcomes Compared to Treatment During Adolescence." Tex Dent J 133(8): 474. | excluded/irrelevant | title |
| Sharma, D., et al. (2014). "Cat eye syndrome." BMJ Case Rep 2014. | excluded/irrelevant | title |
| Tan, R., et al. (2013). CBCT evaluation of changes in the hanges pharyngeal airway in subjects from seven to eighteen years. Computed Tomography: New Research: 394-400. | excluded/irrelevant | title |
| Garnett, B. S., et al. (2019). "Cephalometric comparison of adult anterior open bite treatment using clear aligners and fixed appliances." Angle Orthod 89(1): 3-9. | excluded/irrelevant | title |
| Kirtane, R. S., et al. (2023). "Cephalometric effects of Twin-block and van Beek Headgear-Activator in the correction of Class II malocclusion." Am J Orthod Dentofacial Orthop 163(5): 677-689. | excluded/irrelevant | title |
| Rask, H., et al. (2021). "Cephalometric evaluation of changes in vertical dimension and molar position in adult non-extraction treatment with clear aligners and traditional fixed appliances." Dental Press J Orthod 26(4): e2119360. | excluded/irrelevant | title |
| Bimalrag, B. R., et al. (2024). "Cephalometric Evaluation of the Pre- and Posttreatment Changes after the Correction of Class II Division 1 Malocclusion with Twin Block Appliance in Mixed Dentition." Int J Clin Pediatr Dent 17(7): 783-789. | excluded/irrelevant | title |
| Yassaei, S., et al. (2007). "Changes of tongue position and oropharynx following treatment with functional appliance." J Clin Pediatr Dent 31(4): 287-290. | excluded/irrelevant | title |
| Wu, Q., et al. (2024). "Characterization of condylar movements during mandibular advancement treatment in adolescents with mandibular retrognathism." Chinese Journal of Orthodontics 31(2): 72-77. | excluded/irrelevant | title |
| Verstraete, L., et al. (2020). "Chin wing osteotomy in a patient with craniofrontonasal dysplasia." Oral and Maxillofacial Surgery Cases 6(3). | excluded/irrelevant | title |
| McFarlane, B. (2013). "Class II correction prior to orthodontics with the carriere distalizer." International journal of orthodontics (Milwaukee, Wis.) 24(3): 35-36. | excluded/irrelevant | title |
| Thiesen, G. (2022). "Class II correction with aligners: how to optimize treatment with and without the use of mini-implants anchorage." Revista Clinica de Ortodontia Dental Press 21(2): 58-72. | excluded/irrelevant | title |
| Lombardo, L., et al. (2022). "Class II correction with Carriere Motion 3D Appliance and clear aligner therapy." J Clin Orthod 56(3): 187-193. | excluded/case report | title |
| Awad, S. and M. M. Sadek (2022). "Cephalometric evaluation of the short-term skeletal, dental and soft tissue changes in growing subjects with class II division 1 malocclusion treated with Invisalign® mandibular advancement." Ain Shams Dental Journal (Egypt) 25(1): 63-73 | excluded/case series | full text |
| Horani, S., et al. (2021). "Changes in Airway Dimensions Following Non-extraction Clear Aligner Therapy in Adult Patients with Mild-to-moderate Crowding." J Contemp Dent Pract 22(3): 224-230. | excluded/irrelevant | Title |
| Chen, H., et al. (2023). "Changes of maxillary central incisor and alveolar bone in Class II Division 2 nonextraction treatment with a fixed appliance or clear aligner: A pilot cone-beam computed tomography study." Am J Orthod Dentofacial Orthop 163(4): 509-519. | excluded/irrelevant | title |
| Schupp, W., et al. (2010). "Class II correction with the Invisalign system." J Clin Orthod 44(1): 28-35. | excluded/irrelevant | title |
| Greco, M. and A. Rombolà (2022). "[Class II extraction treatment with aligners: a reliable approach]." Orthod Fr 93(2): 187-204. | excluded/irrelevant | title |
| Patterson, B. D., et al. (2021). "Class II malocclusion correction with Invisalign: Is it possible?" Am J Orthod Dentofacial Orthop 159(1): e41-e48. | excluded/irrelevant | abstract |
| Rongo, R., et al. (2022). "Class II Malocclusion in Adult Patients: What Are the Effects of the Intermaxillary Elastics with Clear Aligners? A Retrospective Single Center One-Group Longitudinal Study." Journal of Clinical Medicine 11(24). | excluded/irrelevant | title |
| Lombardo, L., et al. (2018). "Class II subdivision correction with clear aligners using intermaxilary elastics." Progress in Orthodontics 19(1). | excluded/irrelevant | title |
| Cremonini, F., et al. (2022). "Class II Treatment in Growing Patients: Preliminary Evaluation of the Skeletal and Dental Effects of a New Clear Functional Appliance." Applied Sciences (Switzerland) 12(11). | excluded/ patients were not treated with Precision Wings | abstract |
| Arreghini, A., et al. (2014). "Class II treatment with the Runner in adolescent patients: Combining Twin Block efficiency with aligner aesthetics." Journal of the World Federation of Orthodontists 3(2): e71-e79. | excluded/ patients were not treated with Precision Wings | abstract |
| Gurgel, M. L., et al. (2023). "Clear aligner mandibular advancement in growing patients with Class II malocclusion." AJO DO Clin Companion 3(2): 93-109. | excluded/case series | abstract |
| Wang, X. and J. Gao (2024). "Clear aligner treatment assisted by mini screw for an adult with Class II division 2 malocclusion and a right upper canine completely outside of the dental arch: A case report." Int Orthod 22(1): 100837. | excluded/case report | title |
| Zhang, H., et al. (2024). "Clear aligner treatment of an adult open bite with bilateral missing mandibular first molars through molar protraction with Albert cantilever arms." Int Orthod 22(4): 100918. | excluded/irrelevant | title |
| de Miranda, S. L., et al. (2023). "Clear aligners combined with orthognathic surgery: a case series." General Dentistry 71(1): 66-70. | excluded/case series | title |
| Inchingolo, A. D., et al. (2024). "Clear Aligners in the Growing Patient: A Systematic Review." Children 11(4). | excluded/systematic review | title |
| Raffaini, M. and C. Pisani (2013). "Clinical and cone-beam computed tomography evaluation of the three-dimensional increase in pharyngeal airway space following maxillo-mandibular rotation-advancement for Class II-correction in patients without sleep apnoea (OSA)." Journal of Cranio-Maxillofacial Surgery 41(7): 552-557. | excluded/irrelevant | title |
| Shi, X., et al. (2022). "[Clinical efficacy and influencing factors of molar distalization with clear aligner]." Zhonghua Kou Qiang Yi Xue Za Zhi 57(7): 762-768. | excluded/irrelevant | title |
| Shao, J., et al. (2021). "Clinical efficacy of clear aligner in non-extraction treatment of Angle Class II division 2 malocclusion." Chinese Journal of Orthodontics 28(2): 61-67. | excluded/case series | abstract |
| Meade, M. J. and T. Weir (2024). "Clinical efficacy of the Invisalign mandibular advancement appliance: A retrospective investigation." Am J Orthod Dentofacial Orthop 165(5): 503-512. | excluded/case series | full text |
| Hansa, I., et al. (2020). "Clinical outcomes and patient perspectives of Dental Monitoring® GoLive® with Invisalign®—a retrospective cohort study." Progress in Orthodontics 21(1). | excluded/irrelevant | title |
| Cassetta, M., et al. (2020). "The combined use of clear aligners and computer-guided piezocision: a case report with a 2-year follow-up." Int J Comput Dent 23(1): 57-71. | excluded/irrelevant | title |
| Palone, M., et al. (2023). "Combined use of F22 aligners and intermaxillary elastics in mild skeletal Class II: Two case reports." Journal of Orthodontics 50(1): 77-85. | excluded/ patients were not treated with Precision Wings | title |
| Irsheid, R., et al. (2024). "Comparative assessment of the clinical outcomes of clear aligners compared to fixed appliance in class II malocclusion." Clinical Oral Investigations 28(8). | excluded/irrelevant | title |
| Steele, B. P., et al. (2022). "A comparative assessment of the dentoskeletal effects of clear aligners vs miniplate-supported posterior intrusion with fixed appliances in adult patients with anterior open bite. A multicenter, retrospective cohort study." Am J Orthod Dentofacial Orthop 162(2): 214-228.e214. | excluded/irrelevant | title |
| Vicéns, J. and A. Russo (2010). "Comparative use of Invisalign by orthodontists and general practitioners." Angle Orthod 80(3): 425-434. | excluded/irrelevant | title |
| Isrctn (2020). "Comparing three removable orthodontic devices and no orthodontic treatment in children with excessive overjet." https://trialsearch.who.int/Trial2.aspx?TrialID=ISRCTN12775595. | excluded/study registration | abstract |
| He, J., et al. (2024). "Comparison between clear aligners and twin-block in treating class II malocclusion in children: a retrospective study." J Clin Pediatr Dent 48(5): 125-130. | excluded/patients were not treated with Precision Wings | full text |
| Cretella Lombardo, E., et al. (2024). "Comparison between twin block appliance and mandibular advancement on clear aligners in the improvement of airway dimension: incremental versus maximum bite advancement." Front Oral Health 5: 1463416. | excluded/study did not evaluate Class II correction | full text |
| Griffith, M., et al. (2021). "Comparison of 2 Invisalign tray generations using the Peer Assessment Rating index." Am J Orthod Dentofacial Orthop 160(5): 718-724. | excluded/irrelevant | abstract |
| Sun, Z., et al. (2022). "Comparison of cephalometric measurements of the Twin Block and A6 appliances in the treatment of Class II malocclusion: a retrospective comparative cohort study." Ann Transl Med 10(16): 876. | excluded/patients were not treated with Precision Wings | full text |
| Li, Z. X., et al. (2023). "Comparison of clear aligners and customized lingual appliance for bimaxillary dentoalveolar protrusion: a retrospective study." Shanghai Kou Qiang Yi Xue / Shanghai Journal of Stomatology 32(5): 480-484. | excluded/irrelevant | abstract |
| Fujiyama, K., et al. (2022). "Comparison of clinical outcomes between Invisalign and conventional fixed appliance therapies in adult patients with severe deep overbite treated with nonextraction." Am J Orthod Dentofacial Orthop 161(4): 542-547. | excluded/irrelevant | title |
| Yue, Z., et al. (2023). "Comparison of invisalign mandibular advancement and twin-block on upper airway and hyoid bone position improvements for skeletal class II children: a retrospective study." BMC Oral Health 23(1): 661. | excluded/study did not evaluate Class II correction | full text |
| Hosseini, H. R., et al. (2024). "A comparison of skeletal and dental changes in patients with a Class II relationship treated with clear aligner mandibular advancement and Herbst appliance followed by comprehensive orthodontic treatment." Am J Orthod Dentofacial Orthop 165(2): 205-219. | included |  |
| Camcı, H. and F. Salmanpour (2022). "Comparison of skeletal and dentoalveolar effects of two different mandibular advancement methods: Conventional technique vs aesthetic approach." European Oral Research 56(2): 96-101. | excluded/ patients were not treated with Precision Wings | abstract |
| (2024). Comparison of Skeletal Effects of Herbst and Invisalign MA in Growing Patients With KLass Malocclusion. | excluded/study registration | abstract |
| Tctr (2020). "A Comparison of the Effects of Molar Intrusion between Fixed Appliances with Temporary Anchorage Devices and Clear Aligner on Volumetric Root Resorption and Bite Force in Anterior Open Bite Patients." https://trialsearch.who.int/Trial2.aspx?TrialID=TCTR20201218004. | excluded/study registration | abstract |
| ChiCtr (2021). "Comparison of the treatment effect of two different types of oral appliances in pediatric OSA patients." https://trialsearch.who.int/Trial2.aspx?TrialID=ChiCTR2100051538. | excluded/study registration | abstract |
| Chen, J., et al. (2022). "Comparisons of maxillary incisor retraction effects for patients with first premolar extractions between Damon Q and Invisalign®: A retrospective study." Medicine (Baltimore) 101(40): e30919. | excluded/ patients were not treated with Precision Wings | abstract |
| Boyd, R. L. (2007). "Complex orthodontic treatment using a new protocol for the Invisalign appliance." J Clin Orthod 41(9): 525-547; quiz 523. | excluded/case report | full text |
| Kosyk, M., et al. (2021). "Comprehensive mid-term outcomes following infant mandibular distraction osteogenesis." Cleft Palate-Craniofacial Journal 58(4 SUPPL): 49. | excluded/irrelevant | title |
| Hu, Y. and H. Li (2024). "Concerns about the conclusion that clear aligner mandibular advancement provides better vertical control in growing patients with Class II." Am J Orthod Dentofacial Orthop 166(4): 305-306. | excluded/comment to the authors | full text |
| Selvaraj, A. and A. Kumar Subramanian (2024). "Cone-Beam Computed Tomography Study of Incisive Canal and Maxillary Central Incisors in Dravidian Population." Cureus 16(7): e63707. | excluded/irrelevant | title |
| Vitral, R. W. F., et al. (2023). "Considerations on distalization of maxillary molars with Invisalign aligners in nonextraction patients with Class II malocclusion." American Journal of Orthodontics and Dentofacial Orthopedics 163(3): 295. | excluded/irrelevant | title |
| Huang, A. T. and D. Huang (2022). Controversies in Clear Aligner Therapy: Contemporary Perspectives, Limitations, and Solutions. | excluded/irrelevant | title |
| Kiseri, B., et al. (2018). "The correlation between direction and amount of retrusive movement and condyle position and joint space." Cranio 36(4): 250-256. | excluded/irrelevant | title |
| Waxler, R. (2021). "Correcting skeletal open bite with clear aligners and miniscrews." American Journal of Orthodontics and Dentofacial Orthopedics 160(2): 165. | excluded/irrelevant | title |
| Mampieri, G., et al. (2023). "Correction of Class II malocclusions with aligners: the importance of the treatment staging." Dental Cadmos 91(2): 156-164. | excluded/irrelevant | title |
| Frongia, G. and T. Castroflorio (2012). "Correction of severe tooth rotations using clear aligners: a case report." Aust Orthod J 28(2): 245-249. | excluded/case report | title |
| Tong, B. K., et al. (2020). "CPAP combined with oral appliance therapy reduces CPAP requirements and pharyngeal pressure swings in obstructive sleep apnea." J Appl Physiol (1985) 129(5): 1085-1091. | excluded/irrelevant | title |
| Swennen, G., et al. (2002). "Cranio-facial distraction osteogenesis: a review of the literature. Part II: Experimental studies." Int J Oral Maxillofac Surg 31(2): 123-135. | excluded/irrelevant | title |
| Yáñez-Vico, R. M., et al. (2010). "Craniofacial characteristics in cri-du-chat syndrome." Oral Surgery, Oral Medicine, Oral Pathology, Oral Radiology and Endodontology 110(6): e38-e44. | excluded/irrelevant | title |
| Bowman, S. J., et al. (2015). "Creative adjuncts for clear aligners, part 1: Class II treatment." Journal of clinical orthodontics : JCO 49(2): 83-94. | excluded/case report | full text |
| James Jr, A. E., et al. (1969). "The cri du chat syndrome." Radiology 92(1): 50-52. | excluded/irrelevant | title |
| Lafon, R., et al. (1972). "[Crying cat syndrome discovered during a routine examination]." Ann Med Psychol (Paris) 2(3): 431-438. | excluded/irrelevant | title |
| de Almeida, M. R. (2024). "Current status of the biomechanics of extra-alveolar miniscrews." Journal of the World Federation of Orthodontists 13(1): 25-37. | excluded/irrelevant | title |
| Córdova-Fletes, C., et al. (2012). "A de novo sSMC(22) characterized by high-resolution arrays in a girl with cat-eye syndrome without coloboma." Molecular Syndromology 3(3): 131-135. | excluded/irrelevant | title |
| Blundell, H. L., et al. (2024). "Deep overbite reduction in adolescent patients treated with Invisalign: A retrospective analysis." Am J Orthod Dentofacial Orthop 166(6): 515-523. | excluded/ patients were not treated with Precision Wings | full text |
| Ghazal, A., et al. (2008). "Dental side effects of mandibular advancement appliances - a 2-year follow-up." J Orofac Orthop 69(6): 437-447. | excluded/ patients were not treated with Precision Wings | abstract |
| Lombardo, E. C., et al. (2024). "Dentoskeletal effects of clear aligner vs twin block-a short-term study of functional appliances." J Orofac Orthop 85(5): 317-326. | included |  |
| Kethu, A. (2022). "Development of a smartphone application for Early Diagnosis of Mandibular Retrusion." Sleep Medicine 100: S59. | excluded/irrelevant | title |
| Lin, E., et al. (2022). "Differences in finished case quality between Invisalign and traditional fixed appliances." Angle Orthod 92(2): 173-179. | excluded/irrelevant | title |
| Taffarel, I. A., et al. (2022). "Distalization of maxillary molars with Invisalign aligners in nonextraction patients with Class II malocclusion." Am J Orthod Dentofacial Orthop 162(4): e176-e182. | excluded/ patients were not treated with Precision Wings | abstract |
| Nct (2024). "Distalization With Aligners: anchorage Options." https://clinicaltrials.gov/ct2/show/NCT06631131. | excluded/study registration | title |
| Kravitz, N. D., et al. (2024). "Does age influence deep overbite correction with Invisalign? A prospective study evaluating mandibular incisor intrusion in adolescents vs adults." Angle Orthod 94(2): 145-150. | excluded/ patients were not treated with Precision Wings | abstract |
| Kang, J., et al. (2024). "Does aligner refinement have the same efficiency in deep bite correction?: A retrospective study." BMC Oral Health 24(1): 338. | excluded/ patients were not treated with Precision Wings | abstract |
| Bamagoos, A. A., et al. (2019). "Dose-dependent effects of mandibular advancement on upper airway collapsibility and muscle function in obstructive sleep apnea." Sleep 42(6). | excluded/study did not evaluate Class II correction | abstract |
| Wu, Y., et al. (2023). "Does mandibular advancement with clear aligners have the same skeletal and dentoalveolar effects as traditional functional appliances?" BMC Oral Health 23(1): 65. | included |  |
| Baccetti, T., et al. (2012). "Early vs late orthodontic treatment of deepbite: a prospective clinical trial in growing subjects." Am J Orthod Dentofacial Orthop 142(1): 75-82. | excluded/ patients were not treated with Precision Wings | abstract |
| Elfouly, D., et al. (2024). "The effect of different functional appliances on the sagittal pharyngeal airway dimension in skeletal class II: a retrospective study." Scientific Reports 14(1). | included |  |
| El-Bialy, T. (2020). "The Effect of High-Frequency Vibration on Tooth Movement and Alveolar Bone in Non-Growing Skeletal Class II High Angle Orthodontic Patients: Case Series." Dent J (Basel) 8(4). | excluded/case series | title |
| Xiao, S. L., et al. (2020). "[Effect of invisalign on anterior and posterior upper airway and maxillary bone changes in the treatment of high-angle skeletal Class Ⅱ malocclusion]." Shanghai Kou Qiang Yi Xue 29(4): 410-413. | excluded/case series | abstract |
| Mao, B., et al. (2023). "The effect of maxillary molar distalization with clear aligner: a 4D finite-element study with staging simulation." Prog Orthod 24(1): 16 | excluded/irrelevant | title |
| Al-Worafi, N. A., et al. (2024). "Effect of maxillary molars distalization using clear aligners and fixed orthodontic appliances on the positional and dimensional temporomandibular joint parameters: a three-dimensional comparative study." BMC Oral Health 24(1). | excluded/ patients were not treated with Precision Wings | title |
| Peng, Y. and X. Li (2024). "Effect of molar distalization with clear aligners on occlusal vertical dimension in different vertical craniofacial patterns." Chinese Journal of Tissue Engineering Research 28(28): 4559-4564. | excluded/ patients were not treated with Precision Wings | abstract |
| Muro, M. P., et al. (2023). "Effectiveness and predictability of treatment with clear orthodontic aligners: A scoping review." Int Orthod 21(2): 100755. | excluded/review | title |
| Yan, X., et al. (2023). "Effectiveness of clear aligners in achieving proclination and intrusion of incisors among Class II division 2 patients: a multivariate analysis." Prog Orthod 24(1): 12. | excluded/case series | abstract |
| Kau, C. H., et al. (2017). "Effectiveness of Clear Aligners in Treating Patients with Anterior Open Bite: A Retrospective Analysis." Journal of clinical orthodontics : JCO 51(8): 454-460. | excluded/study did not evaluate Class II correction | title |
| Al-Jewair, T., et al. (2020). "Effects of clear aligner therapy for Class II malocclusion on upper airway morphology and daytime sleepiness in adults: A case series." Int Orthod 18(1): 154-164. | excluded/case series | title |
| Moradinejad, M., et al. (2024). "Effects of clear aligners on the vertical position of the molar teeth and the vertical and sagittal relationships of the face: a preliminary retrospective before-after clinical trial." BMC Oral Health 24(1): 234. | excluded/study did not evaluate Class II correction | title |
| D’Antò, V., et al. (2024). "Effects of clear aligners treatment in growing patients: a systematic review." Frontiers in Oral Health 5. | excluded/systematic review | title |
| Yoshimoto, H., et al. (2013). "Effects of distraction osteogenesis on the inferior alveolar nerve of the cat mandible." Journal of Oral and Maxillofacial Surgery, Medicine, and Pathology 25(3): 201-204. | excluded/irrelevant | title |
| Henick, D., et al. (2021). "Effects of Invisalign (G5) with virtual bite ramps for skeletal deep overbite malocclusion correction in adults." Angle Orthod 91(2): 164-170. | excluded/patients were not treated with Precision Wings | abstract |
| Pogal-Sussman-Gandia, C. B., et al. (2019). "Effects of Invisalign(®) treatment on speech articulation." Int Orthod 17(3): 513-518. | excluded/ irrelevant | title |
| Nakano, M., et al. (2009). "Effects of mandibular advancement on growth after condylectomy." J Dent Res 88(3): 261-265. | excluded/ irrelevant | title |
| Lione, R., et al. (2022). "Effects of pendulum appliance versus clear aligners in the vertical dimension during Class II malocclusion treatment: a randomized prospective clinical trial." BMC Oral Health 22(1). | excluded/ irrelevant | title |
| Zhu, C., et al. (2022). "Effects of the advanced mandibular spring on mandibular retrognathia treatment: a three-dimensional finite element study." BMC Oral Health 22(1): 271. | excluded/ irrelevant | title |
| Burashed, H. (2023). "The efficacy of anterior open bite closure when using Invisalign's optimized extrusion versus conventional attachments." J World Fed Orthod 12(3): 112-117. | excluded/ irrelevant | title |
| Kong, L. and X. Q. Liu (2023). "Efficacy of invisible advancement correction for mandibular retraction in adolescents based on Pancherz analysis." World J Clin Cases 11(6): 1299-1309. | excluded/case series | full text |
| Martin Romero, M., et al. (2013). "Efficacy of orthoapnea mandibular advance device in patients with obstructive sleep apnoea syndrome." American Journal of Respiratory and Critical Care Medicine 187. | excluded/irrelevant | abstract |
| Saif, B. S., et al. (2022). "Efficiency evaluation of maxillary molar distalization using Invisalign based on palatal rugae registration." Am J Orthod Dentofacial Orthop 161(4): e372-e379. | excluded/irrelevant | title |
| Auladell, A., et al. (2022). "The efficiency of molar distalization using clear aligners and mini-implants: Two clinical cases." International Orthodontics 20(1). | excluded/case series | title |
| Liaw, J., et al. (2022). "En-masse retraction of maxillary anterior teeth with the Double J retractor and palatal miniscrews: A case report." Am J Orthod Dentofacial Orthop 161(4): 592-601. | excluded/case report | title |
| Boyd, R. L. (2008). "Esthetic orthodontic treatment using the invisalign appliance for moderate to complex malocclusions." J Dent Educ 72(8): 948-967. | excluded/irrelevant | title |
| Taneja, I. Z., et al. (2024). "Evaluating the efficiency of mandibular molar protraction using Herbst appliances versus temporary anchorage devices: a retrospective case-controlled study." Prog Orthod 25(1): 32. | excluded/irrelevant | title |
| Akbulut, A. and D. D. Kilinç (2019). "Evaluation of condyle position in patients with Angle Class I, II, and III malocclusion using cone-beam computed tomography panoramic reconstructions." Oral Radiology 35(1): 43-50. | excluded/irrelevant | title |
| Wu, Q., et al. (2024). "Evaluation of mandibular motion in adolescents with skeletal class II division 1 malocclusion during mandibular advancement using clear functional aligners: a prospective study." BMC Oral Health 24(1): 320. | excluded/ study did not evaluate Class II correction | full text |
| Mirzasoleiman, P., et al. (2024). "Evaluation of Mandibular Projection in Class II Division 2 Subjects Following Orthodontic Treatment Using Clear Aligners." Journal of Contemporary Dental Practice 25(4): 295-302. | excluded/patients were not treated with Precision Wings | full text |
| Tabrizi, R., et al. (2015). "Evaluation of Mandibular Wing Osteotomy in Obstructive Sleep Apnea Cases with Retrognathia." Journal of Maxillofacial and Oral Surgery 14(1): 46-50. | excluded/irrelevant | title |
| Feng, Q., et al. (2024). "Evaluation of modified clear Twin Block aligner in treating adolescents with skeletal class II malocclusion: A two-centre cephalometric study." Orthod Craniofac Res 27(4): 665-673. | excluded/patients were not treated with Precision Wings | abstract |
| Harris, K., et al. (2020). "Evaluation of open bite closure using clear aligners: a retrospective study." Prog Orthod 21(1): 23. | excluded/ study did not evaluate Class II correction | abstract |
| Giannasi, L. C., et al. (2019). "Evaluation of the masticatory muscle function, physiological sleep variables, and salivary parameters after electromechanical therapeutic approaches in adult patients with Down syndrome: a randomized controlled clinical trial." Trials 20(1): 215. | excluded/irrelevant | title |
| Grave, K. and G. Townsend (2005). "Evaluation of the outcomes of 7 Class II treatments 40 years later." World J Orthod 6(4): 331-342. | excluded/irrelevant | title |
| Bowman, E., et al. (2023). "Evaluation of the predicted vs. achieved occlusal outcomes with the Invisalign® appliance: A retrospective investigation of adult patients." Int Orthod 21(2): 100746. | excluded/irrelevant | title |
| Zehairy, S. A. A., et al. (2024). "Evaluation of the skeletal and dental effects of a hybrid aesthetic functional appliance (HAF) in skeletal class II division 1 malocclusion: A prospective uncontrolled clinical trial." J Dent Res Dent Clin Dent Prospects 18(1): 55-62. | excluded/patients were not treated with Precision Wings | abstract |
| Chang, C. H., et al. (2022). "Excessive overjet, steep mandibular plane, and posterior buccal crossbite treated with aligners, infrazygomatic crest bone screws, and Class II elastics." AJO-DO Clinical Companion 2(6): 512-522. | excluded/case report | abstract |
| Antelo, O. M., et al. (2021). "Extraction Treatment of Class II, Division 2 Malocclusion and Deep Overbite Using Aligners and Temporary Anchorage Devices." Journal of Clinical Orthodontics 55(1): 59-68. | excluded/irrelevant | title |
| Giancotti, A., et al. (2006). "Extraction treatment using Invisalign Technique." Prog Orthod 7(1): 32-43. | excluded/irrelevant | title |
| Chen, X., et al. (2023). "Factors influencing the efficacy of invisalign in molar distalization and tooth movement." Front Bioeng Biotechnol 11: 1215169. | excluded/irrelevant | title |
| Mosca, A. L., et al. (2007). "Fortuitous FISH diagnosis of an interstitial microdeletion (5)(q31.1q31.2) in a girl suspected to present a cri-du-chat syndrome." American Journal of Medical Genetics, Part A 143(12): 1342-1347. | excluded/irrelevant | title |
| Rabie, A. B., et al. (2003). "Functional appliance therapy accelerates and enhances condylar growth." Am J Orthod Dentofacial Orthop 123(1): 40-48. | excluded/irrelevant | title |
| Liu, C., et al. (2022). "Functional clear aligner technique in the treatment of class II malocclusion in juvenile: A case report and literature review." Journal of Radiation Research and Applied Sciences 15(3): 59-64. | excluded/case report | title |
| Li, Z., et al. (2019). "Functional clear aligner treatment of class Ⅱ malocclusion in teenagers." Hua Xi Kou Qiang Yi Xue Za Zhi / West China Journal of Stomatology 37(3): 236-241. | excluded/patients were not treated with Precision Wings | full text |
| Greco, M., et al. (2022). "G-Block: Posterior anchorage device tads-supported after molar distalization with aligners: An adult case report." International Orthodontics 20(4). | excluded/case report | title |
| Quintero-Rivera, F. and J. A. Martinez-Agosto (2013). "Hemifacial microsomia in cat-eye syndrome: 22q11.1-q11.21 as candidate loci for facial symmetry." Am J Med Genet A 161a(8): 1985-1991. | excluded/irrelevant | title |
| El-Fateh, T. and S. Ruf (2011). "Herbst treatment with mandibular cast splints--revisited." Angle Orthod 81(5): 820-827. | excluded/patients were not treated with Precision Wings | title |
| Dai, F., et al. (2024). "How accurate is predicted root movement achieved in four first-premolar extraction cases with Invisalign?" Orthod Craniofac Res 27(6): 985-995. | excluded/patients were not treated with Precision Wings | title |
| Al-Worafi, N. A., et al. (2024). "Impact of molar teeth distalization by clear aligners on maxillary alveolar bone thickness and root resorption: a three‑dimensional study." BMC Oral Health 24(1): 237. | excluded/irrelevant | title |
| Al-Tayar, B., et al. (2023). "Impact of molar teeth distalization by clear aligners on temporomandibular joint: a three-dimensional study." Prog Orthod 24(1): 25. | excluded/patients were not treated with Precision Wings | title |
| Caruso, S., et al. (2019). "Impact of molar teeth distalization with clear aligners on occlusal vertical dimension: a retrospective study." BMC Oral Health 19(1): 182. | excluded/patients were not treated with Precision Wings | title |
| Staderini, E., et al. (2020). "Indication of clear aligners in the early treatment of anterior crossbite: A case series." Dental Press Journal of Orthodontics 25(4): 33-43. | excluded/case series | title |
| Deli, R., et al. (2008). "Individual effects of functional therapy in growing patients." Mondo Ortodontico 33(4): 219-227. | excluded/patients were not treated with Precision Wings | abstract |
| Husain, F., et al. (2024). "Influence of Invisalign precision bite ramp utilization on deep bite correction and root length in adults." Angle Orthod 94(5): 488-495. | excluded/irrelevant | title |
| Miao, Z., et al. (2023). "Influence of maxillary molar distalization with clear aligners on three-dimensional direction: molar distal movement, intrusion, distal tip and crown buccal torque." Prog Orthod 24(1): 48. | excluded/irrelevant | title |
| Wieslander, L. (1984). "Intensive treatment of severe Class II malocclusions with a headgear-Herbst appliance in the early mixed dentition." Am J Orthod 86(1): 1-13. | excluded/irrelevant | title |
| Schwestka-Polly, R., et al. (2000). "Introduction of "vario plates" for retention after mandibular distraction osteogenesis." Int J Adult Orthodon Orthognath Surg 15(4): 283-289. | excluded/irrelevant | title |
| Zybutz, T., et al. (2021). "Investigation and comparison of patient experiences with removable functional appliances." Angle Orthod 91(4): 490-495. | excluded/ study did not evaluate Class II correction | full text |
| Schwartz, B. (2012). "Invisalign and aesthetic dentistry." N Y State Dent J 78(4): 36-37. | excluded/irrelevant | title |
| Shin, K. (2017). "The Invisalign Appliance Could Be an Effective Modality for Treating Overbite Malocclusions Within a Mild to Moderate Range." J Evid Based Dent Pract 17(3): 278-280. | excluded/irrelevant | title |
| Miller, D. B. (2009). "Invisalign in TMD treatment." Int J Orthod Milwaukee 20(3): 15-19. | excluded/irrelevant | title |
| Voudouris, J. C., et al. (2018). "Invisalign mandibular advancers and tetrahedron 3D facial analysis in dentofacial orthopedics: 10 rules." J Clin Orthod 52(3): 134-147. | excluded/irrelevant | title |
| Mampieri, G. and A. Giancotti (2013). "Invisalign technique in the treatment of adults with pre-restorative concerns." Prog Orthod 14(1): 40. | excluded/irrelevant | title |
| Fischer, K. (2010). "Invisalign treatment of dental Class II malocclusions without auxiliaries." J Clin Orthod 44(11): 665-672; quiz 687. | excluded/patients were not treated with Precision Wings | abstract |
| Sabouni, W., et al. (2022). "Invisalign treatment with mandibular advancement: A retrospective cohort cephalometric appraisal." J Clin Imaging Sci 12: 42. | excluded/case series | full text |
| Dickerson, T. E. (2017). "Invisalign with Photobiomodulation: Optimizing Tooth Movement and Treatment Efficacy with a Novel Self-Assessment Algorithm." J Clin Orthod 51(3): 157-165. | excluded/irrelevant | title |
| Meier, B., et al. (2003). "Invisalign--patient profiling. Analysis of a prospective survey." J Orofac Orthop 64(5): 352-358. | excluded/irrelevant | title |
| Schupp, W., et al. (2010). "Invisalign(®) treatment of patients with craniomandibular disorders." Int Orthod 8(3): 253-267. | excluded/irrelevant | title |
| (2024). Invisalign® System with Mandibular Advancement Featuring Occlusal Blocks (MAOB) Post-Market Study. | excluded/study registration | abstract |
| Malik, O. H., et al. (2013). "Invisible orthodontics part 1: invisalign." Dent Update 40(3): 203-204, 207-210, 213-205. | excluded/irrelevant | title |
| Albertini, E., et al. (2025). "Invisible treatment with preadjusted lingual appliance and monolateral space opening for an adult Class II malocclusion with upper lateral incisors agenesis: An ortho-prosthetic case report." International Orthodontics 23(1). | excluded/case report | title |
| Xi, T., et al. (2020). "Landmark-Based Versus Voxel-Based 3-Dimensional Quantitative Analysis of Bimaxillary Osteotomies: A Comparative Study." Journal of Oral and Maxillofacial Surgery 78(3): 468.e461-468.e410. | excluded/irrelevant | title |
| Dumars, K. W., et al. (1964). "LE CRI DU CHAT ( CRYING CAT ) SYNDROME." American Journal of Diseases of Children 108(5): 533-+ | excluded/irrelevant | title |
| Gupta, A., et al. (2017). "The long-term effects of mandibular advancement splint on cardiovascular fitness and psychomotor performance in patients with mild to moderate obstructive sleep apnea: a prospective study." Sleep Breath 21(3): 781-789. | excluded/irrelevant | title |
| Li, Q. and K. Yang (2024). "Loss of attachments in patients during orthodontic therapy with clear aligners: A prospective clinical study." Orthod Craniofac Res 27(2): 244-250. | excluded/irrelevant | title |
| Giancotti, A., et al. (2015). "Lower incisor extraction treatment with the Invisalign® technique: three case reports." J Orthod 42(1): 33-44. | excluded/case series | title |
| Gudhimella, S., et al. (2022). "Management of Anterior Open Bite and Skeletal Class II Hyperdivergent Patient with Clear Aligner Therapy." Turk J Orthod 35(2): 139-149. | excluded/irrelevant | title |
| Khosravi, R., et al. (2017). "Management of overbite with the Invisalign appliance." Am J Orthod Dentofacial Orthop 151(4): 691-699.e692. | excluded/irrelevant | title |
| El-Bialy, T. (2021). "Mandibular Advancement in Adult Skeletal Class II Patients Using Clear Aligners and Photobiomodulation." Journal of Clinical Orthodontics 55(1): 11-19. | excluded/irrelevant | title |
| Mohammadieh, A. M., et al. (2022). "Mandibular Advancement Splint Therapy." Adv Exp Med Biol 1384: 373-385. | excluded/irrelevant | abstract |
| Yu, L., et al. (2023). "Mandibular advancement with clear aligners and functional appliances in the treatment of skeletal Class Ⅱ malocclusion: a systematic review and meta-analysis." Hua Xi Kou Qiang Yi Xue Za Zhi 41(3): 305-314. | excluded/systematic review | title |
| Caruso, S., et al. (2021). "Mandibular advancement with clear aligners in the treatment of skeletal Class II. A retrospective controlled study." Eur J Paediatr Dent 22(1): 26-30. | included |  |
| Andriola, F. O., et al. (2024). "Mandibular autorotation: a critical virtual parameter in clinical decision-making regarding maxilla-first versus mandible-first sequence." International Journal of Oral and Maxillofacial Surgery 53(8): 698-706. | excluded/irrelevant | title |
| Debras, P., et al. (2024). "Mandibular osteotomy, a simpler and safer method of osteosynthesis." Revue medicale de Liege 79(2): 99-103. | excluded/irrelevant | title |
| Kiliaridis, S., et al. (2010). "Masseter muscle thickness as a predictive variable in treatment outcome of the twin-block appliance and masseteric thickness changes during treatment." Orthod Craniofac Res 13(4): 203-213. | excluded/irrelevant | title |
| Li, L., et al. (2023). "Maxillary molar distalization with a 2-week clear aligner protocol in patients with Class II malocclusion: A retrospective study." Am J Orthod Dentofacial Orthop 164(1): 123-130. | excluded/irrelevant | title |
| Azami, N., et al. (2022). "Maxillomandibular advancement with a "surgery first" approach and Invisalign for treatment of obstructive sleep apnea." J Clin Orthod 56(1): 49-58. | excluded/irrelevant | title |
| Jiang, Q., et al. (2021). "Mesialization of mandibular molars with clear aligners in a young class II, division 2 patient." J Clin Orthod 55(10): 617-626. | excluded/irrelevant | title |
| Liu, F., et al. (2024). "Miniscrew anchorage versus Class II elastics for maxillary arch distalization using clear aligners." Angle Orthod 94(4): 383-391. | excluded/irrelevant | title |
| Giancotti, A., et al. (2014). "A miniscrew-supported intrusion auxiliary for open-bite treatment with Invisalign." J Clin Orthod 48(6): 348-358. | excluded/irrelevant | title |
| Marchiori Farret, M., et al. (2008). "Molar changes with cervical headgear alone or in combination with rapid maxillary expansion." Angle Orthod 78(5): 847-851. | excluded/irrelevant | title |
| Tagliaro, M. L., et al. (2009). "Morphological changes in the mandible of male mice associated with aging and biomechanical stimulus." Anat Rec (Hoboken) 292(3): 431-438. | excluded/irrelevant | title |
| Bedoya, A., et al. (2014). "Morphometry of the cranial base and the cranial-cervical-mandibular system in young patients with type II, division 1 malocclusion, using tomographic cone beam." Cranio 32(3): 199-207. | excluded/irrelevant | title |
| Labarca, G., et al. (2022). "Mouth Closing to Improve the Efficacy of Mandibular Advancement Devices in Sleep Apnea." Ann Am Thorac Soc 19(7): 1185-1192. | excluded/irrelevant | title |
| Liu, J., et al. (2022). "A multifactorial intervention to increase adherence to oral appliance therapy with a titratable mandibular advancement device for obstructive sleep apnea: a randomized controlled trial." Sleep Breath 26(4): 1739-1745. | excluded/irrelevant | abstract |
| Tctr (2023). "Myofunctional therapy with clear aligner treament in anterior open bite pateint." https://trialsearch.who.int/Trial2.aspx?TrialID=TCTR20230908003. | excluded/study registration | abstract |
| Cremonini, F., et al. (2024). "A New Functional Approach to Class II Treatment with Clear Aligners in Growing Patients." Journal of Clinical Orthodontics 58(4): 221-235. | excluded/case report | abstract |
| Lombardo, L., et al. (2024). "Night-Time 3D-Printed Aligners and Intermaxillary Elastics for Treatment of an Adolescent Class II Subdivision Patient." Journal of Clinical Orthodontics 58(7): 418-426. | excluded/case report | abstract |
| Robertson, L. J. and T. El-Bialy (2022). "Non-surgical Treatment of a Late Adolescent Patient with Skeletal Class II Malocclusion Using Clear Aligners: A Case Report." Open Dentistry Journal 16(1). | excluded/case report | title |
| Arveda, N., et al. (2024). "Non-surgical treatment of a severe deep bite with aligners and miniscrew: A hybrid approach." APOS Trends in Orthodontics 14(2): 130-138. | excluded/irrelevant | title |
| Chamberland, S. and N. Nataf (2024). "Noninvasive conservative management of anterior open bite treated with TADs versus clear aligner therapy." Clin Oral Investig 28(4): 236. | excluded/irrelevant | title |
| Costa, T. S., et al. (2024). "Nonsurgical Treatment of an Adult Skeletal Class II Malocclusion with Clear Aligners." Journal of Clinical Orthodontics 58(6): 354-365. | excluded/irrelevant | title |
| Singh, B. P., et al. (2024). "Occlusal interventions for managing temporomandibular disorders." Cochrane Database of Systematic Reviews(9). | excluded/irrelevant | title |
| Mohamed, A. M., et al. (2024). "Oral appliance therapy vs. positional therapy for managing positional obstructive sleep apnea; a systematic review and meta-analysis of randomized control trials." BMC Oral Health 24(1): 666. | excluded/systematic review | title |
| Okawara, Y., et al. (2004). "Oral appliance titration and nasal resistance in nonapneic subjects." Am J Orthod Dentofacial Orthop 126(5): 620-622. | excluded/irrelevant | title |
| Veldhuis, S. K., et al. (2015). "Oral appliance to assist non-invasive ventilation in a patient with amyotrophic lateral sclerosis." Sleep Breath 19(1): 61-63. | excluded/irrelevant | title |
| Knowles, S., et al. (2023). "Oral Appliances for OSA Treatment: Meeting the Quadruple Aim." Mil Med 188(3-4): e718-e724. | excluded/irrelevant | title |
| Mickelson, S. A. (2020). "Oral Appliances for Snoring and Obstructive Sleep Apnea." Otolaryngol Clin North Am 53(3): 397-407. | excluded/irrelevant | title |
| Martín Romero, M., et al. (2013). "Oral appliances in obstructive sleep apnea syndrome: Our experience." Sleep Medicine 14: e247-e248. | excluded/irrelevant | title |
| Eshky, R. T. (2022). "Orthodontic correction of Class II skeletal malocclusion complicated by deep bite using clear aligners: A case report." J Taibah Univ Med Sci 17(6): 962-968. | excluded/case report | title |
| Koukou, M., et al. (2022). "Orthodontic Management of Skeletal Class II Malocclusion with the Invisalign Mandibular Advancement Feature Appliance: A Case Report and Review of the Literature." Case Rep Dent 2022: 7095467. | excluded/case report | title |
| Ngan, P. and S. K. Tai (2024). "Orthopedic treatment of Class II malocclusion with mandibular deficiency: a clinical practice review." Frontiers of Oral and Maxillofacial Medicine 6. | excluded/review | title |
| Thereza-Bussolaro, C., et al. (2019). "Pharyngeal dimensional changes in class II malocclusion treatment when using Forsus® or intermaxillary elastics - An exploratory study." Int Orthod 17(4): 667-677 | excluded/irrelevant | title |
| Meade, M. J. and T. Weir (2024). "Planned and achieved overjet and overbite changes following an initial series of Invisalign® aligners: A retrospective study of adolescent patients." Int Orthod 22(3): 100888. | excluded/irrelevant | abstract |
| Nct (2016). "Posterior Segment Intrusion Using Miniplates." https://clinicaltrials.gov/show/NCT02674191. | excluded/irrelevant | title |
| Greco, M. and A. Rombolà (2022). "Precision bite ramps and aligners: An elective choice for deep bite treatment." Journal of Orthodontics 49(2): 213-220. | excluded/irrelevant | title |
| Blundell, H. L., et al. (2023). "Predictability of anterior open bite treatment with Invisalign." Am J Orthod Dentofacial Orthop 164(5): 674-681. | excluded/irrelevant | title |
| Lim, Z. W., et al. (2023). "The predictability of maxillary curve of Wilson leveling with the Invisalign appliance." Journal of the World Federation of Orthodontists 12(5): 207-212 | excluded/irrelevant | title |
| D’Antò, V., et al. (2023). "Predictability of Maxillary Molar Distalization and Derotation with Clear Aligners: A Prospective Study." International Journal of Environmental Research and Public Health 20(4). | excluded/irrelevant | title |
| Ren, L., et al. (2022). "The predictability of orthodontic tooth movements through clear aligner among first-premolar extraction patients: a multivariate analysis." Prog Orthod 23(1): 52. | excluded/irrelevant | title |
| Blundell, H. L. D., et al. (2021). "Predictability of overbite control with the Invisalign appliance." Am J Orthod Dentofacial Orthop 160(5): 725-731. | excluded/irrelevant | abstract |
| Blundell, H. L., et al. (2022). "Predictability of overbite control with the Invisalign appliance comparing SmartTrack with precision bite ramps to EX30." Am J Orthod Dentofacial Orthop 162(2): e71-e81. | excluded/irrelevant | title |
| Shahabuddin, N., et al. (2023). "Predictability of the deep overbite correction using clear aligners." Am J Orthod Dentofacial Orthop 163(6): 793-801. | excluded/irrelevant | abstract |
| Meade, M. J. and T. Weir (2024). "Predicted and achieved overjet and overbite measurements with the Invisalign appliance: a retrospective study." Angle Orthod 94(1): 3-9. | excluded/irrelevant | abstract |
| Meade, M. J., et al. (2024). "Predicted overbite and overjet changes with the Invisalign appliance: a validation study." Angle Orthod 94(1): 10-16. | excluded/irrelevant | abstract |
| Lu, W., et al. (2023). "Preformed intrusion bulbs on clear aligners facilitate active vertical control in a hyperdivergent skeletal Class II case with extraction: A case report with 4-year follow-up." APOS Trends in Orthodontics 13(1): 46-54. | excluded/case report | title |
| Hönn, M. and G. Göz (2006). "A premolar extraction case using the Invisalign system." J Orofac Orthop 67(5): 385-394. | excluded/case report | title |
| Glaser, B. J., et al. (2022). "Prospective multicenter investigation of Invisalign treatment with the mandibular-advancement feature: An interim report." J Clin Orthod 56(8): 458-463. | excluded/case series | full text |
| Austin, D. F., et al. (2010). "A protocol for improved stability with Herbst appliance treatment for adults." Prog Orthod 11(2): 151-156. | excluded/irrelevant | title |
| Janssens, Y., et al. (2024). "Quality of occlusal outcome in adult Class II patients treated with completely customized lingual appliances and Class II elastics compared to adult Class I patients." European Journal of Orthodontics 46(5). | excluded/irrelevant | title |
| Burashed, H. and R. E. Sebai (2023). "Quantifying the efficacy of overbite reduction in patients treated with clear aligners using optimized versus conventional attachments." J World Fed Orthod 12(3): 105-111. | excluded/irrelevant | abstract |
| Zhao, Z., et al. (1999). "[Quantitative evaluation of new bone formation in the posterior zone of the glenoid fossa in response to the mandibular advancement in rats]." Hua Xi Kou Qiang Yi Xue Za Zhi 17(2): 152-154. | excluded/irrelevant | title |
| Rai, P. and R. Sobti (2024). "Queries regarding maxillary molar distalization with a 2-week clear aligner protocol in patients with Class II malocclusion." Am J Orthod Dentofacial Orthop 165(6): 609-610. | excluded/irrelevant | title |
| Hennessy, J., et al. (2016). "A randomized clinical trial comparing mandibular incisor proclination produced by fixed labial appliances and clear aligners." Angle Orthod 86(5): 706-712. | excluded/irrelevant | title |
| Vardimon, A. D., et al. (1998). "Rapid palatal expansion. Part 2: Dentoskeletal changes in cats with patent versus synostosed midpalatal suture." American Journal of Orthodontics and Dentofacial Orthopedics 113(5): 488-497. | excluded/irrelevant | title |
| Nct (2020). "RCT Comparing Invisalign and Traditional Orthodontic Treatment." https://clinicaltrials.gov/show/NCT04556448. | excluded/study registration | abstract |
| Kinzinger, G. S. M., et al. (2018). "A retrospective cephalometric investigation of two fixed functional orthodontic appliances in class II treatment: Functional Mandibular Advancer vs. Herbst appliance." Clin Oral Investig 22(1): 293-304. | excluded/patients were not treated with Precision Wings | full text |
| Atole, S., et al. (2024). "Revolutionizing Class II Division 1 Malocclusion Treatment With Forsus Appliance: A Clinical Case." Cureus 16(8): e66930. | excluded/case report | title |
| Zhang, L., et al. (2022). "Risk factors for midcourse correction during treatment of first series of aligners with Invisalign." Am J Orthod Dentofacial Orthop 162(2): e96-e102. | excluded/ study did not evaluate Class II correction | abstract |
| Withayanukonkij, W., et al. (2023). "Root resorption during maxillary molar intrusion with clear aligners: a randomized controlled trial." Angle Orthodontist 93(6): 629-637. | excluded/irrelevant | title |
| Pinho, T., et al. (2023). "Scissor Bite in Growing Patients: Case Report Treated with Clear Aligners." Children (Basel) 10(4). | excluded/case report | title |
| Sabouni, W., et al. (2023). "Scope of clear aligner therapy (CAT) in Phase I (early) orthodontic treatment." Seminars in Orthodontics 29(2): 216-236. | excluded/case report | full text |
| Uribe, F., et al. (2011). "A segmented appliance for space closure followed by Invisalign and fixed appliances." Orthodontics (Chic.) 12(4): 386-395. | excluded/irrelevant | title |
| Eckhart, J. E. (2009). "Sequential MARA-Invisalign treatment." J Clin Orthod 43(7): 439-448; quiz 459. | excluded/irrelevant | title |
| Jedraszak, G., et al. (2013). "A severe prenatal presentation of Cat Eye Syndrome." Clinical Dysmorphology 22(4): 175-177. | excluded/irrelevant | title |
| Shen, G. (2015). "[SGTB orthopedic regime to correct protrusive skeletal anomalies: a developmental path through evolution, renovation and innovation]." Shanghai Kou Qiang Yi Xue 24(5): 513-518. | excluded/irrelevant | title |
| Ravera, S., et al. (2021). "Short term dentoskeletal effects of mandibular advancement clear aligners in Class II growing patients. A prospective controlled study according to STROBE Guidelines." Eur J Paediatr Dent 22(2): 119-124. | included |  |
| (2022). Short-term and Long-term Evaluation of Three-dimensional Morphological Condylar and Mandibular Changes in Patients Affected by Juvenile Idiopathic Arthritis Treated With Mandibular Advancement Clear Aligner. A Prospective Controlled Study. C. University of Milan - Prof. Maspero and A. University of Milan - Dr. Abate. | excluded/irrelevant | title |
| Suh, H., et al. (2023). "Short-term stability of anterior open bite treatment with clear aligners in adults." Am J Orthod Dentofacial Orthop 164(6): 774-782. | excluded/irrelevant | title |
| Greco, M., et al. (2021). "Simplifying the approach of open bite treatment with aligners and selective micro-osteoperforations: An adult case report." Int Orthod 19(1): 159-169. | excluded/case report | title |
| Wen, S., et al. (2025). "Skeletal Class II malocclusion management with clear aligners and mini-implants." AJO-DO Clinical Companion 5(1): 56-69. | excluded/irrelevant | abstract |
| Agarwal, Y., et al. (2021). "Skeletal open bite treated with clear aligners and miniscrews." American Journal of Orthodontics and Dentofacial Orthopedics 160(2): 166-167. | excluded/irrelevant | title |
| Xi, T., et al. (2023). "Skeletal structure of asymmetric mandibular prognathism and retrognathism." Maxillofacial Plastic and Reconstructive Surgery 45(1). | excluded/irrelevant | title |
| Huang, A. T. and D. Huang (2018). "Space management with Invisalign for interdisciplinary orthodontic treatment." J Clin Orthod 52(4): 219-226. | excluded/irrelevant | title |
| Huang, W., et al. (2023). "Stress distribution of the modified clear twin-block aligner on the temporomandibular joint, alveolar bone and teeth: A finite element analysis." International Orthodontics 21(4). | excluded/irrelevant | title |
| Xu, B. and H. Urban (1998). "[The study on treatment of Chinese children's Class II malocclusion by Herbst appliance]." Zhonghua Kou Qiang Yi Xue Za Zhi 33(2): 113-115. | excluded/irrelevant | title |
| van der Weijden, F. N., et al. (2024). "Successful Use of Orthodontic Aligners in a Young Brass Instrumentalist A Case Report." Medical Problems of Performing Artists 39(3): 148-154. | excluded/case report | title |
| Bastidas-Castillo, D. A. and P. Ramirez-Naranjo (2024). "Surgery first with clear aligners for a Class II patient: Case report and literature review." J Stomatol Oral Maxillofac Surg 125(2): 101672. | excluded/case report | title |
| Chang, J., et al. (2019). ""Surgery-First" Approach with Invisalign Therapy to Correct a Class II Malocclusion and Severe Mandibular Retrognathism." J Clin Orthod 53(7): 397-404. | excluded/irrelevant | title |
| Womack, W. R. and R. H. Day (2008). "Surgical-orthodontic treatment using the Invisalign system." J Clin Orthod 42(4): 237-245. | excluded/irrelevant | title |
| Li, Y., et al. (2024). "Sustained Protraction Increases Lengthening of the Mandibular Condylar Process Whilst Changes Its Growth Direction in a Rat Model." Orthod Craniofac Res. | excluded/irrelevant | title |
| Corcuera-Flores, J. R., et al. (2016). "A systematic review of the oral and craniofacial manifestations of cri du chat syndrome." Clinical Anatomy 29(5): 555-560. | excluded/irrelevant | title |
| Henrikson, T. and M. Nilner (2003). "Temporomandibular disorders, occlusion and orthodontic treatment." J Orthod 30(2): 129-137; discussion 127. | excluded/irrelevant | title |
| Quinzi, V., et al. (2024). "Teenage Patients with Class II Subdivision Treated with Aligners and Elastics: A Retrospective Study." Medicina (Kaunas) 60(12). | excluded/patients were not treated with Precision Wings | full text |
| Shaeran, T. A. T. and A. R. Samsudin (2019). "Temporomandibular Joint Ankylosis Leading to Obstructive Sleep Apnea." J Craniofac Surg 30(8): e714-e717. | excluded/irrelevant | title |
| Hyoun, S. C., et al. (2012). "Teratogen update: Methotrexate." Birth Defects Research Part A - Clinical and Molecular Teratology 94(4): 187-207. | excluded/irrelevant | title |
| Giancotti, A., et al. (2020). "Thermoformed Retainer: An Effective Option for Long-Term Stability." Case Rep Dent 2020: 8861653. | excluded/irrelevant | title |
| Chen, Y. F., et al. (2021). "Three-Dimensional Analysis of the Condylar Hypoplasia and Facial Asymmetry in Craniofacial Microsomia Using Cone-Beam Computed Tomography." Journal of Oral and Maxillofacial Surgery 79(8): 1750.e1751-1750.e1710. | excluded/irrelevant | title |
| Yamashita, A. L., et al. (2017). "Three-dimensional analysis of the pharyngeal airway space and hyoid bone position after orthognathic surgery." Journal of Cranio-Maxillofacial Surgery 45(9): 1408-1414. | excluded/irrelevant | title |
| Ahmed, M. J., et al. (2024). "Three-dimensional computed tomography analysis of airway volume in growing class II patients treated with Frankel II appliance." Head and Face Medicine 20(1). | excluded/patients were not treated with Precision Wings | title |
| Xie, J., et al. (2023). "Three-dimensional dentoskeletal effects of the Angel align A6 clear aligners in a skeletal Class II growing patient: A case report." Int Orthod 21(2): 100756. | excluded/patients were not treated with Precision Wings | title |
| Nindra, J., et al. (2021). "Three-dimensional evaluation of condyle-glenoid fossa complex following treatment with herbst appliance." Journal of Clinical Medicine 10(20). | excluded/patients were not treated with Precision Wings | title |
| Liebregts, J. H. F., et al. (2015). "Three-dimensional facial simulation in bilateral sagittal split osteotomy: A validation study of 100 patients." Journal of Oral and Maxillofacial Surgery 73(5): 961-970. | excluded/irrelevant | title |
| Zhang, Y., et al. (2024). "Three-dimensional spatial analysis of temporomandibular joint in adolescent Class II division 1 malocclusion patients: comparison of Twin-Block and clear functional aligner." Head and Face Medicine 20(1). | excluded/ study did not evaluate Class II correction | full text |
| Zheng, J., et al. (2023). "Three-dimensional spatial analysis of the temporomandibular joint in adult patients with Class II division 2 malocclusion before and after orthodontic treatment: a retrospective study." BMC Oral Health 23(1): 477. | excluded/irrelevant | title |
| Veys, B., et al. (2017). "Three-dimensional volumetric changes in the upper airway after maxillomandibular advancement in obstructive sleep apnoea patients and the impact on quality of life." International Journal of Oral and Maxillofacial Surgery 46(12): 1525-1532. | excluded/irrelevant | title |
| Schwartz, J. P., et al. (2023). "Tomographic evaluation of changes induced by herbst treatment - buccolingual inclination of mandibular canines and the intercanine distance." Acta Scientiarum - Health Sciences 45. | excluded/irrelevant | title |
| Fan, W. J., et al. (2024). "Transforming Adolescent Smiles: Correcting Skeletal Mandibular Retrusion and Bimaxillary Protrusion with Clear Aligners." Curr Med Sci 44(3): 657-666. | excluded/irrelevant | abstract |
| Hematpour, S., et al. (2014). "Treatment of a unilateral class II malocclusion with Sabbagh Universal Spring: a case report." Int J Orthod Milwaukee 25(2): 9-12. | excluded/case report | title |
| Sun, R. and P. Liu (2022). "Treatment of an adolescent patient with Class II Division 2 malocclusion with mandibular retrognathism and excessive overbite using Invisalign aligners." AJO-DO Clinical Companion 2(6): 572-588. | excluded/case report | abstract |
| Gidarakou, I. K. and V. Tzatzakis (2022). "Treatment of an adolescent special-needs patient using Invisalign's mandibular-advancement protocol." J Clin Orthod 56(8): 487-493. | excluded/case report | title |
| Sabouni, W., et al. (2019). "Treatment of class II for growing patients by clear aligners: which protocol?" L' Orthodontie francaise 90(1): 13-27. | excluded/case series | full text |
| Inchingolo, A. D., et al. (2022). "Treatment of Class III Malocclusion and Anterior Crossbite with Aligners: A Case Report." Medicina (Kaunas) 58(5). | excluded/case report | title |
| Giancotti, A. and A. Farina (2010). "Treatment of collapsed arches using the invisalign system." J Clin Orthod 44(7): 416-425. | excluded/case report | abstract |
| Dianiskova, S., et al. (2022). "Treatment of mild Class II malocclusion in growing patients with clear aligners versus fixed multibracket therapy: A retrospective study." Orthod Craniofac Res 25(1): 96-102. | excluded/patients were not treated with Precision Wings | full text |
| Palone, M., et al. (2023). "Treatment of severe Class II skeletal malocclusion in a hyperdivergent adult patient via hybrid clear aligner approach: A case report." J Orthod 50(2): 205-214. | excluded/case report | title |
| Gasparello, G. G., et al. (2023). "Treatment of severe deep overbite with nonextraction treatment with Invisalign or conventional fixed appliance. Some points to be considered for clinical comparison." Am J Orthod Dentofacial Orthop 163(1): 1-2. | excluded/comments to the authors | abstract |
| Giancotti, A. and R. Di Girolamo (2009). "Treatment of severe maxillary crowding using Invisalign and fixed appliances." J Clin Orthod 43(9): 583-589; quiz 582. | excluded/irrelevant | title |
| Pavoni, C., et al. (2022). "Treatment Timing Considerations for Mandibular Advancement with Clear Aligners in Skeletal Class II Malocclusions." Journal of Clinical Orthodontics 56(8): 464-471. | excluded/irrelevant | title |
| Baccetti, T., et al. (2000). "Treatment timing for Twin-block therapy." Am J Orthod Dentofacial Orthop 118(2): 159-170. | excluded/irrelevant | title |
| Sadek, M. M. and R. Alhashmi (2024). "Unplanned tooth movement in deepbite correction with Invisalign: A retrospective study." J World Fed Orthod 13(3): 136-144. | excluded/irrelevant | title |
| Hillman, D. R., et al. (2003). "The upper airway during anaesthesia." British Journal of Anaesthesia 91(1): 31-39. | excluded/irrelevant | title |
| Ojima, K., et al. (2018). "Upper molar distalization with Invisalign treatment accelerated by photobiomodulation." J Clin Orthod 52(12): 675-683. | excluded/irrelevant | title |
| Nct (2021). "Upper Sequential Distalization With TADs and Aligners." https://clinicaltrials.gov/show/NCT04875104. | excluded/study registration | abstract |
| Harnick, D. J. (2012). "Using clear aligner therapy to correct malocclusion with crowding and an open bite." Gen Dent 60(3): 218-223. | excluded/irrelevant | title |
| Xi, T., et al. (2013). "Validation of a novel semi-automated method for three-dimensional surface rendering of condyles using cone beam computed tomography data." International Journal of Oral and Maxillofacial Surgery 42(8): 1023-1029. | excluded/irrelevant | title |
| Miller, R. J., et al. (2003). "Validation of Align Technology's Treat III digital model superimposition tool and its case application." Orthod Craniofac Res 6 Suppl 1: 143-149. | excluded/irrelevant | title |
| Nct (2022). "Vertical Effects in Class II Patients Treated With Distalization." https://clinicaltrials.gov/show/NCT05298280. | excluded/study registration | abstract |
| Balboni, A., et al. (2023). "Vertical effects of distalization protocol with Clear aligners in Class II patients: a prospective study." Minerva Dental and Oral Science 72(6): 291-297. | excluded/irrelevant | title |
| Yaghoutiazar, Sahar; Yadegari, Atiye; Esmaeili, Saharnaz; Hajizadeh, Alireza; Shahbazi, Soheil. Functional Class II Treatment Simultaneous with Selective Reduction of Talon Cusps: A Case Report  Clinical Case Reports; Bognor Regis Vol. 12, Ed. 12, (Dec 1, 2024). DOI:10.1002/ccr3.70015 | excluded/case report | title |
| Koaban Abdullah; Al-Harbi, Sahar K; Al-Shehri, Abdulrahman Z; Al-Shamri, Buthainah S; Aburazizah, Maha F; et al. Current Trends in Pediatric Orthodontics: A Comprehensive Review. Cureus; Palo Alto Vol. 16, Ed. 9, (2024). DOI:10.7759/cureus.68537 | excluded/review | title |
| Huang, Anderson T, DDS; Huang, Darren, DDS. Two-phase Treatment with a Growth Regulator and Clear Aligner Therapy of a Class II Adolescent Patient. New York State Dental Journal; Hempstead Vol. 87, Ed. 6, (Nov 2021): 14-20. | excluded/case report | abstract |
| Align Technology Inc (ALGN). GlobalData Company Profiles - Medical Devices Pipeline Summary; London, (Apr 2019). | excluded/irrelevant | title |
| Align Technology Inc – 10K or Int'l Equivalent, 2023. Reportal; Kuching, Vol. Aa-Ca, (2023). | excluded/irrelevant | title |
| Align Technology Announces New Teen Solution with Introduction of Invisalign Teen With Mandibular Advancement: First clear aligner to simultaneously align teeth and reposition the jaw in growing patients. Marketwired; Toronto (Mar 6, 2017). | excluded/irrelevant | title |
| Clinical Pearl: The Importance of the “Pre-MA” Phase. Werner, Alison.  Orthodontic Products (Online); Los Angeles: Anthem Media Group. (Feb 14, 2019) | excluded/irrelevant | title |
| Clear Aligner Technique. Tai, Sandra. Batavia, US, Batavia: Quintessence Publishing Co, Oct 28, 2019. | excluded/irrelevant | title |
| Temporary Anchorage Devices in Clinical Orthodontics. US: John Wiley & Sons, Incorporated, Apr 21, 2020. | excluded/irrelevant | title |
| Invisalign Cleared for Treatment with Mandibular Advancement. Werner, Alison.  Orthodontic Products (Online); Los Angeles: Anthem Media Group. (Oct 29, 2018) | excluded/irrelevant | title |
| Aligner Techniques in Orthodontics. Palma Moya, Susana; Lozano Zafra, Javier. GB: John Wiley & Sons, Incorporated, Jun 14, 2021. | excluded/irrelevant | title |
| Press Release: Align Technology Announces Invisalign(R) System with Mandibular Advancement Featuring Occlusal Blocks for Class II Skeletal and Dental Correction. Dow Jones Institutional News; New York. 01 Apr 2025. | excluded/irrelevant | title |
| Align Technology Announces Invisalign® System with Mandibular Advancement Featuring Occlusal Blocks for Class II Skeletal and Dental Correction. ICT Monitor Worldwide, Amman. 02 Apr 2025. | excluded/irrelevant | title |
| Align Technology Inc Investor Day – Final. Fair Disclosure Wire; Linthicum. 06 Sep 2023. | excluded/irrelevant | title |
| Press Release: Align Technology Receives 510(k) Clearance from the FDA For Invisalign(R) Treatment with Mandibular Advancement in the U.S. Dow Jones Institutional News; New York. 29 Oct 2018. | excluded/irrelevant | title |
| Q1 2022 Align Technology Inc Earnings Call – Final. Fair Disclosure Wire; Linthicum. 27 Apr 2022. | excluded/irrelevant | title |
| Event Brief of Q1 2022 Align Technology Inc Earnings Call – Final. Fair Disclosure Wire; Linthicum. 28 Apr 2022. | excluded/irrelevant | title |
| Align Technology Unveils Invisalign Teen with Mandibular Advancement. Professional Services Close - Up; Jacksonville. 09 Mar 2017. | excluded/irrelevant | title |
| Press Release: Align Technology Announces New Teen Solution with Introduction of Invisalign Teen with Mandibular Advancement. Dow Jones Institutional News; New York. 06 Mar 2017. | excluded/irrelevant | title |
